# An imbalance between CD80 and CD86 levels and CD4 regulatory T cell number and transendocytosis function exists in the liver in autoimmune hepatitis

**DOI:** 10.1093/cei/uxag013

**Published:** 2026-03-11

**Authors:** Neil Halliday, Alan Kennedy, Cayman Williams, Blagoje Soskic, Claudia Hinze, Massimo Pinzani, Douglas Thorburn, David M Sansom

**Affiliations:** Institute for Liver and Digestive Health, University College London, London, UK; Institute of Immunity and Transplantation, University College London, London, UK; Institute of Immunity and Transplantation, University College London, London, UK; Institute of Immunity and Transplantation, University College London, London, UK; Institute of Immunity and Transplantation, University College London, London, UK; Institute of Immunity and Transplantation, University College London, London, UK; Institute for Liver and Digestive Health, University College London, London, UK; UPMC-ISMETT, Palermo, Italy; Institute for Liver and Digestive Health, University College London, London, UK; Institute of Immunity and Transplantation, University College London, London, UK

**Keywords:** autoimmune hepatitis, Treg, CD80, CD86, transendocytosis, CD28 costimulation

## Abstract

Impairment in Regulatory CD4 T cells (Treg) number and function have been implicated in autoimmune hepatitis (AIH). Treg are critical regulators of CD28 costimulation through CTLA4-mediated CD80 and CD86 control. We sought evidence for hepatic Treg frequency, phenotype, and function, and CD80/CD86 availability in AIH. Hepatic immune cells were isolated from eight patients with AIH and compared with cirrhotic and non-cirrhotic controls. Cells were assessed by flow cytometry and function was assessed by acquisition of CD80 from model antigen-presenting cells (APCs). We observed that Treg frequency was increased in AIH liver. Treg had an activated phenotype with a high CTLA4 expression and higher frequency of CTLA4 + PD1+ cells compared to non-AIH. Conventional CD4 T cells (Tcon) had an activated phenotype with increased HLA-DR expression, despite patients being in biochemical and histological remission. CD80 and CD86 expressions on B cells and monocytes were maintained or increased despite the excess of Treg, suggesting an imbalance between Treg and CD28 ligand availability. Hepatic Treg in AIH had preserved function for transendocytosis of CD80, which was enhanced by IL2 or IL10, demonstrating capacity for CD28 control. Overall, hepatic Treg have an activated phenotype and we did not observe reduced frequency or transendocytosis function of Treg in cirrhotic liver or (sub)acute liver failure from AIH. However, there is an imbalance between Treg function and CD80 and CD86 availability, with Tcon activation. This suggests that advanced AIH is not associated with reduced Treg frequency or function in the liver, but with an excess of CD80 and CD86.

## Teaser

Autoimmune hepatitis (AIH) is a rare, T cell mediated liver disease that can result in acute liver and progressive chronic liver disease. We have described the phenotype of immune cells from within the liver of patients with AIH and demonstrate preserved Treg frequency with an activated phenotype and preserved functional capacity to transendocytose CD80. However, CD80 and CD86 levels were maintained suggesting an imbalance between Treg function and CD80 and CD86 availability and therefore possibly of CD28 costimulation.

## Graphical Abstract

**Graphical Abstract:**
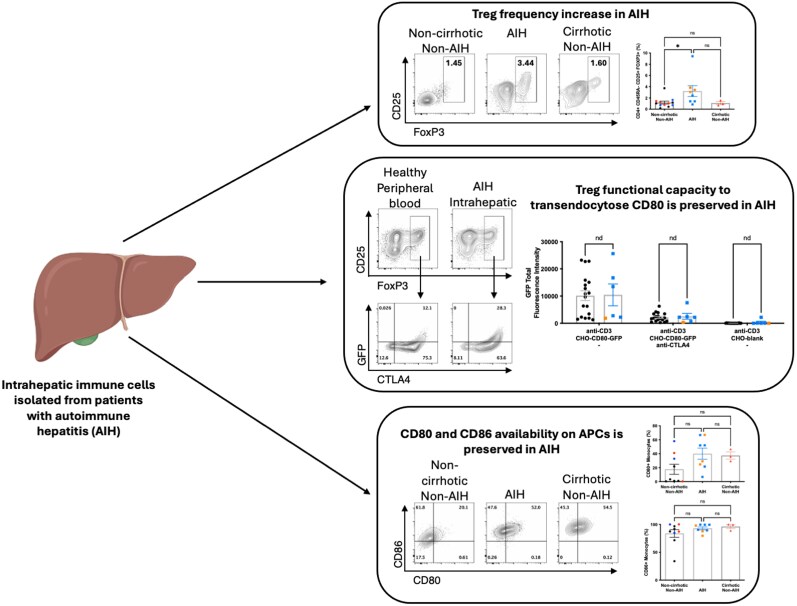

## Introduction

Autoimmune hepatitis (AIH) is a disorder that results in parenchymal liver inflammation and can result in acute liver failure or chronic liver disease resulting in cirrhosis [1]. AIH is characterized by genetic associations with variants in class I and class II HLA and immune regulatory genes, including cytotoxic T lymphocyte antigen 4 (CTLA4) and SH2B3 [2, 3]. Patients with AIH typically have circulating autoantibodies, including anti-nuclear, anti-smooth muscle, and anti–liver–kidney microsomal antibodies and raised polyclonal immunoglobulin G [1]. Treatment is conventionally with immunosuppressive medications, although advanced chronic liver disease and acute liver failure may require liver transplantation (LT).

Regulatory CD4 T cells (Treg) have been implicated in the pathogenesis of AIH, with evidence for Treg dysfunction, impaired Treg frequency, and an imbalance in Treg:T helper (Th)17 ratios in peripheral blood [4–8]. CD25 + FoxP3+ Treg are critical for the maintenance of self-tolerance by restraining autoreactive immune responses by various effector mechanisms [9, 10]. Loss or deficiency of Treg results in widespread autoimmunity, including AIH-like syndromes, in both animal models and humans [11–13].

The associations of Treg and genetic polymorphisms in CTLA4, including an enhancer variant that results in low CTLA4 expression [3], with the onset and progression of AIH suggest that alterations in the control of CD28 costimulation by Treg may underlie the pathogenesis of AIH. CTLA4 is a critical effector for Treg control of autoimmune responses by regulation of the ligands for CD28. CD28 provides the dominant costimulation signal for CD4 and CD8 T cells following engagement with the ligands CD80 or CD86, which are expressed on APCs [14], and uniquely to the liver, on the liver sinusoidal endothelium [15] and hepatocytes [16, 17]. CTLA4 is a high-affinity receptor for CD80 and CD86, resulting in the suppression of CD28 signals by competitive binding and removal and destruction of CD80 and CD86 by transendocytosis [18]. CTLA4 is constitutively expressed by Treg, and loss of CTLA4 in animal models and human haploinsufficiency results in lymphoproliferation, solid organ inflammation, and autoimmunity, including liver pathology [19]. Similarly, direct CD28 agonism has been shown to induce an AIH-like phenotype in a mouse model [20], thus supporting a model where defective Treg or CTLA4 function leads to excessive CD28 costimulation, T cell activation, and autoimmunity within the liver.

Studies of Treg number, phenotype, and function in AIH have mainly relied upon the examination of peripheral blood Treg from patients, which may not reflect the biology occurring at the site of inflammation. We therefore undertook an assessment of the intrahepatic Treg and conventional CD4 T cell (Tcon) (non-Treg CD4 T cells) populations from explanted liver tissue at the time of LT, compared with liver tissue from noncirrhotic, non-AIH liver (as a proxy for ‘healthy’ liver) and from cirrhotic liver tissue from non–immune-mediated liver disease. Defining the immune perturbations in AIH has the potential to identify new biomarkers of disease activity and prognosis, to explore new treatment targets, and to enhance our understanding of the pathogenesis of autoimmune liver conditions. Here, we report changes in the frequency and phenotype of Treg, evidence for chronic activation of Tcon, and preserved or elevated levels of CD80 and CD86, suggesting a possible imbalance in Treg and control of CD80 and CD86 in AIH. *Ex vivo* testing of intrahepatic Treg demonstrated preserved capacity for activation and CD80 transendocytosis, suggesting that Treg retain functional capability with respect to control of ligands of CD28 in AIH.

## Materials and methods

### Patient samples

Patients with a diagnosis of AIH based on a modified International Autoimmune Hepatitis Group (IAIHG) score of 6 or more, undergoing liver transplantation, were included [21]. Explanted livers were collected in phosphate-buffered saline (PBS) and, after clinical pathological assessment, excess liver tissue was processed for study. AIH liver samples included those with cirrhosis and those undergoing LT for (sub)acute liver failure, representing severe or end-stage AIH disease. Due to the rarity of samples, both acute and chronic AIH samples were combined for analysis.

Noncirrhotic, non-AIH liver tissue was obtained from several sources, including (i) livers offered as donor organs for transplantation but not used for logistical or clinical indications (e.g. extensive vascular or capsular injury on retrieval), (ii) liver perfusion fluid from donor-transplanted livers (which provides a population of immune cells reflective of the intrahepatic immune environment [22]), and (iii) distal margins from liver resections of colorectal metastases from patients without background liver disease. Cirrhotic, non-AIH tissue was obtained from explanted livers from patients with alcohol-related liver disease, who were abstinent in line with transplant guidance, undergoing liver transplantation for chronic liver disease.

### Isolation of intrahepatic mononuclear immune cells

Liver tissue was prepared by washing twice in ice-cold PBS, followed by maceration in 0.001% bovine pancreas DNase 1 (Sigma-Aldrich) and 0.01% type IV collagenase (ThermoFisher Scientific) containing Hanks’ Balanced Salt Solution with added calcium, magnesium, and phenol red (Life Technologies). Macerated tissue was incubated with regular agitation at 37°C for 30 minutes. Cells were collected after straining macerated tissue through a metal sieve, with the breakdown of residual tissue by pressure through the sieve, followed by a 70-μm cell strainer (Fisher Scientific). The cell suspension was diluted in Roswell Park Memorial Institute media (RPMI) (Invitrogen) containing 10% fetal calf serum (LabTech), 1% penicillin and streptomycin (Invitrogen), and 2 mM glutamine (Sigma-Aldrich) (complete RPMI). Samples were centrifuged for 2 minutes at 50 × *g* to remove hepatocytes, then layered 1:1:1 over 40% isotonic Percoll (GE Healthcare), Ficoll-Paque PLUS (GE Healthcare) gradients, and centrifuged at 1060 × *g* for 25 minutes without brake. Mononuclear cells were collected from the interface and resuspended in complete RPMI or in 0.5% bovine serum albumin and 2 mM EDTA in PBS (MACS buffer) at 1 × 10^8^ cells/ml for negative selection of CD4 T cells.

Immune cells from transplant perfusion fluid were isolated by centrifugation at 750 × *g* for 15 minutes, followed by density gradient centrifugation over Ficoll-Paque PLUS at 400 × *g* for 20 minutes without a brake. The mononuclear cell layer was collected and washed with PBS and then centrifuged for 15 minutes at 750 × *g* and resuspended in complete RPMI.

### Peripheral blood mononuclear cell isolation

Healthy donor peripheral blood was obtained from leukodepletion cones (NHS Blood and Transplant) or from fresh blood draws from healthy donors. Leukocyte cones were diluted 1:6, and fresh blood 1:2 with PBS and mononuclear cells were isolated by density gradient centrifugation over Ficoll-Paque PLUS gradients following centrifugation at 1060 × *g* for 25 minutes without brake. The mononuclear cell layer was retrieved and washed using PBS with centrifugation for 10 minutes at 1060 × *g*, 5 minutes at 60 × *g* and 5 minutes at 490 × *g*. Mononuclear cells were resuspended in MACS buffer at 1 × 10^8^ cells/ml for CD4 T cell–negative selection.

### CD4T cell isolation

Total CD4 T cells were isolated from liver or peripheral blood mononuclear cell populations by immunomagnetic negative selection using the EasySep Human CD4+ T cell Enrichment Kit (StemCell Technologies), as per the manufacturer’s instructions. Briefly, mononuclear cells in MACS buffer were incubated with 50 μl of enrichment cocktail per 1 × 10^8^ cells for 10 minutes at room temperature. Following this, 100 μl EasySep D Magnetic Particles were added per 1 × 10^8^ cells and incubated for 5 minutes at room temperature. The cells were transferred to EasySep magnets for 5 minutes, and non–bound cells were poured off for collection, washed twice in PBS, and then resuspended in complete RPMI. Purity assessed by flow cytometry routinely exceeded 95% CD4 T cells.

### Flow cytometry

Multicolour flow cytometry was performed on isolated cells after staining with Fixable Viability Dye eF780 (eBiosciences) and surface staining with anti-CD3 V500 (clone UCHT1; BD Biosciences), anti-CD4 AF700 (RPA-T4; BD Biosciences), anti-CD25 BV605 (2A3; BD Biosciences), anti-CD45RA Percp-Cy5.5 (HI100; eBiosciences), anti-CD39 PE-Cy7 (A1; Biolegend), anti-ICOS (CD278) BUV395 (DX29; BD Biosciences), anti-PD1 (CD279) BV786 (EH12.2HZ; BD Biosciences), anti-HLA-DR FITC (G46-6; BD Biosciences), anti-CD28 PE-Cy7 (CD28.2; BD Biosciences), anti-CD14 AF700 (M5E2; BD Biosciences), anti-CD86 BV421 (IT2.2; Biolegend), anti-CD80 PE (L307.4; BD Biosciences), and anti-CD19 BV786 (SJ25C1; BD Biosciences). Staining of intracellular antigens was performed after fixation and permeabilization using the FoxP3 Staining Kit (eBiosciences) with anti-FoxP3 APC (236A/E7; eBiosciences), anti-Ki67 FITC (B56; BD Biosciences), and anti-CTLA4 (CD152) PE (BNI3; BD Biosciences).

Flow cytometry was performed with an LSRFortessa (BD Biosciences) and analysed using FlowJo version 10.8.1 (Treestar). The gating strategy to identify populations of interest is shown in Supplementary Figure S1. Variation in fluorescence intensity between runs was controlled for by the use of Application Settings at the time of acquisition to account for drift in cytometer settings. To further ensure standardization for variation in baseline autofluorescence from different tissue preparations, relative median fluorescence intensity (MFI) was calculated. Within each sample, a recognized population of cells known not to express the marker of interest was selected, and the MFI of the positive population was expressed as a ratio of the MFI of the negative population. This standardization to an internal negative population reduces variation further. Negative and positive populations were defined based on the known internal negative population (Supplementary Figure S1c).

### CD80 transendocytosis assays

In total, 2 × 10^5^ freshly isolated CD4 T cells from peripheral blood or liver tissue were co-cultured in complete RPMI with 1 μg/ml anti-CD3 (OKT3; BioXCell) for 16 hours alongside Chinese Hamster Ovary (CHO) cells either transduced to express human CD80 labelled with a C-terminal Green Fluorescent Protein (GFP) tag (CHO-CD80) or not (CHO-Blank) at 1:1 ratio. Anti-CTLA4 (ticilimumab was a gift from Pfizer) was added at 20 μg/ml, and recombinant human interleukin (IL) 2 or IL10 (both Peprotech) was added at stated concentrations with or without 20 μg/ml anti-CD210 (anti-IL10RA) (clone 3F9, BD Biosciences), as stated. CD80 acted as both a CD28 costimulatory ligand for activation and a target for transendocytosis. Cells were collected, and GFP acquisition was assessed by flow cytometry. GFP acquisition was determined as the total fluorescence intensity (TFI) of GFP, defined as the frequency of GFP-containing FoxP3+ Treg multiplied by the mean GFP fluorescence intensity of GFP-containing Treg.

### Statistical analysis

Statistical analysis was conducted using Prism version 10.10 (GraphPad Software). One-way analysis of variance (ANOVA) test was used when comparing multiple groups of continuous variables, with Tukey’s multiple comparison tests of significance. Independent t tests were applied to compare the means of independent continuous variables for GFP TFI. Statistical significance was indicated by *P* values ≤0.05.

## Results

### Patient cohort

Liver explant samples were obtained from eight patients undergoing orthotopic liver transplantation for liver failure due to AIH. Seven patients were female with an average age at transplantation of 38.5 years (range: 25–57 years). Three patients did not have evidence of chronicity on histological examination of their explant and were indicated for transplantation for (sub)acute liver failure. The remaining five patients had cirrhosis with end-stage liver disease, of whom four met the criteria for complete biochemical response (ALT and immunoglobulin G within the normal laboratory range) at the time of transplantation [23], and all five had minimal inflammatory activity in the explanted liver. All patients except for one were taking immunosuppressive medications at the time of transplantation. Patient clinical characteristics, biochemistry, and explant histological findings are summarized in Table 1.

**Table 1:** Demographic and clinical characteristics of included patients with autoimmune hepatitis.

Age (years)	Gender	Modified disease score	Serum autoantibodies^b^			Medication^a^	ALT^a^ (IU/ml)	IgG^a^ (g/l)	Bilirubin^a^ (μmol/l)	INR	Clinical presentation	Explant histology^a^
			ANA	aSMA	aLKM							
47	F	6	+++	−	−	Aza, Pred	20	na	25	1.2	Cirrhotic type 1 AIH	Cirrhosis, minimal interface hepatitis
42	F	8	na	+++	−	Tac, Aza, Pred	309	40	380	2.4	Subacute fulminant AIH	Severe acute hepatitis, panacinar necrosis
25	F	6	na	−	+	MP, Pred	44	6.1	34	1.2	Cirrhotic type 1 AIH	Cirrhosis, no interface hepatitis, mild lymphocytic infiltrate
32	F	6	na	−	++	Aza, Pred	27	12.2	34	1.2	Cirrhotic type 2 AIH	Cirrhosis, no interface hepatitis, mild lymphocytic infiltrate
33	F	6	na	Na	na	Aza	16	10	78	1.2	Cirrhotic type 1 AIH	Cirrhosis, no interface hepatitis, mild lymphocytic infiltrate
27	M	7	+	−	−	HC, Tac	284	18.3	478	1.7	Cirrhotic type 1 AIH	Cirrhosis, no interface hepatitis, mild lymphocytic infiltrate
57	F	8	1:160	1:80	—	Aza, Pred	227	8.6	480	1.2	Subacute fulminant AIH	Bridging necrosis, mild lymphocytic infiltrate
45	F	7	1:320	—	—	Nil	1898	22.6	684	3.1	Acute fulminant AIH	Lobular necroinflammation, lymphoplasmacytic infiltrate

AIH, Autoimmune Hepatitis; aLKM, anti-liver kidney microsomal antibody; ALT, alanine aminotransferase; ANA, antinuclear antibody; aSMA, anti-smooth muscle antibody; Aza, Azathioprine; HC, hydrocortisone; IgG, immunoglobulin G; INR, international normalized ratio; MP, mercaptopurine; na, not available; Pred, prednisolone; Tac, tacrolimus.

^a^At time of transplantation.

^b^Where titer is not stated, reported if >1:100 with +weak, ++ medium, +++ strong, ++++ very strong (>1:1000).

### Intrahepatic Treg frequency is higher in AIH

**Figure 1:**
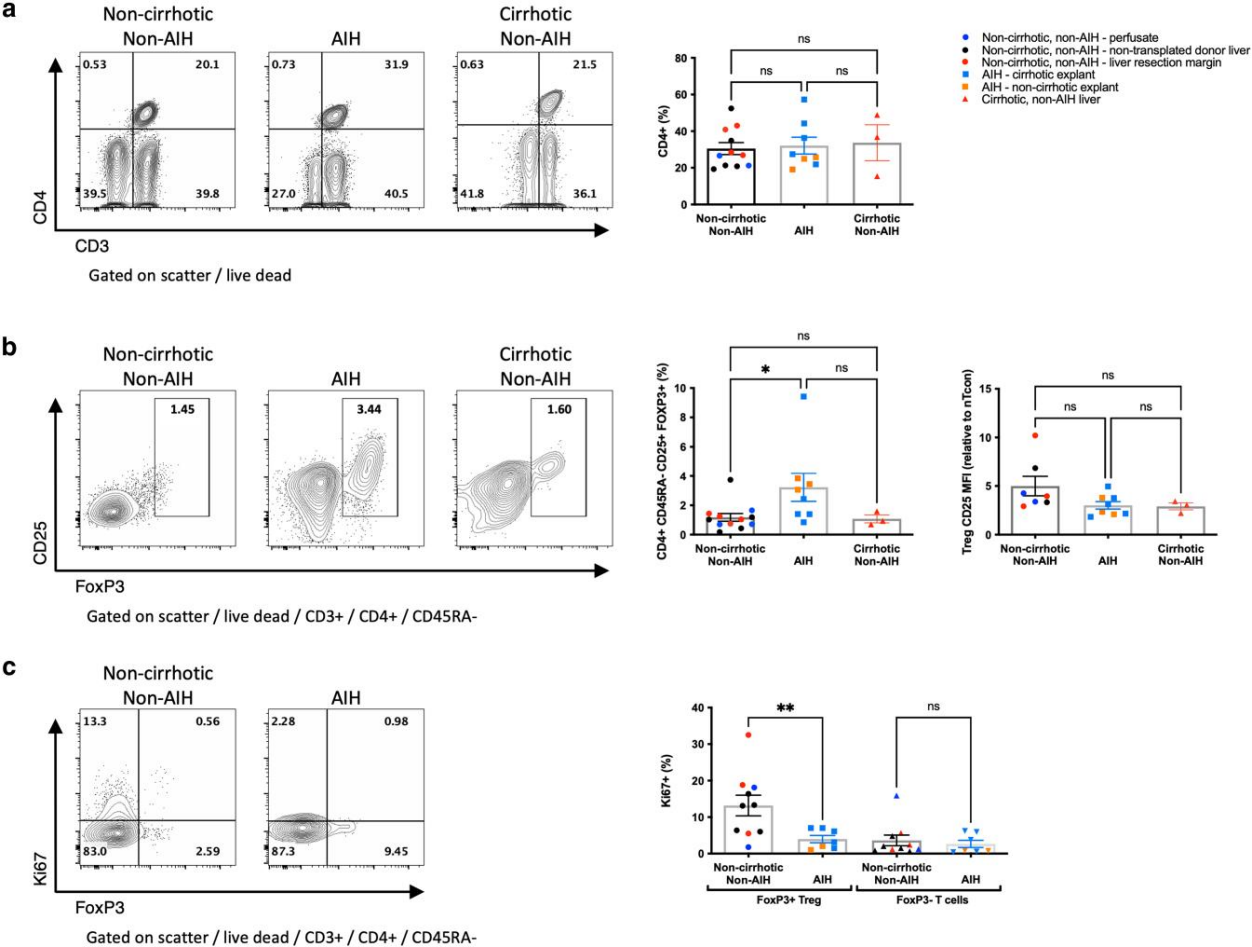
Comparison of T cell frequency and proliferation in liver tissue. a: Comparison of CD4+ T cell frequency within the CD3+ T lymphocyte population, comparing liver tissue from patients with AIH to those without. b: Comparison of Treg frequency, demonstrating the percentage of CD4 + CD45RA-CD25 + FoxP3+ within CD3+ T lymphocyte population, illustrates a higher Treg frequency in AIH liver tissue compared to non-AIH liver tissue. c: Ki67 expression was lower in Treg from AIH liver tissue compared with non-AIH liver tissue, but similar when comparing Tcon. Flow cytometry plots show a representative sample from each group, graphs show aggregate data for all samples tested. ns, non-significant, * *P* < 0.05, ** *P* < 0.01 by one way ANOVA with Tukey’s multiple comparison test or by unpaired *t*-test for comparisons of 3 or 2 groups, respectively.

We first examined the frequency of Treg in the intrahepatic mononuclear cell population isolated from all three groups of liver tissue. We observed that the frequency of CD4 + CD45RA− memory T cells within the CD3+ T cell population was the same in non-AIH noncirrhotic, cirrhotic, and AIH liver (Fig. 1a). However, CD4 + CD45RA-CD25 + FoxP3+ Treg frequency, as a percentage of CD3 T cells, was significantly higher in AIH liver compared with noncirrhotic, non-AIH liver and cirrhotic liver (3.3%, 1.18%, and 1.08%, respectively, *P* < 0.05) (Fig. 1b). Within the Treg populations, CD25 expression was similar in all groups (Fig. 1b), although Ki67 expression was significantly lower in AIH Treg compared with noncirrhotic, non-AIH liver Treg (3.98% vs 13.19%, *P* < 0.01) (Fig. 1c). However, Ki67 expression was similar in Tcon in both groups (3.63 vs 2.65%, *P* = 0.9) (Fig. 1c). Subanalysis, separating (sub)acute AIH from cirrhotic AIH, showed similar trends; although a statistically significant increase in Treg frequency in AIH was not observed, the trend remained (Supplementary Figure S2). Taken together, these observations suggested a preserved or higher frequency of Treg in the liver in AIH compared with both non-AIH chronic liver disease and non-cirrhotic, non-AIH liver, despite relatively low levels of cell proliferation.

### Intrahepatic Treg in AIH have enhanced the expression of CTLA4 and PD1

**Figure 2:**
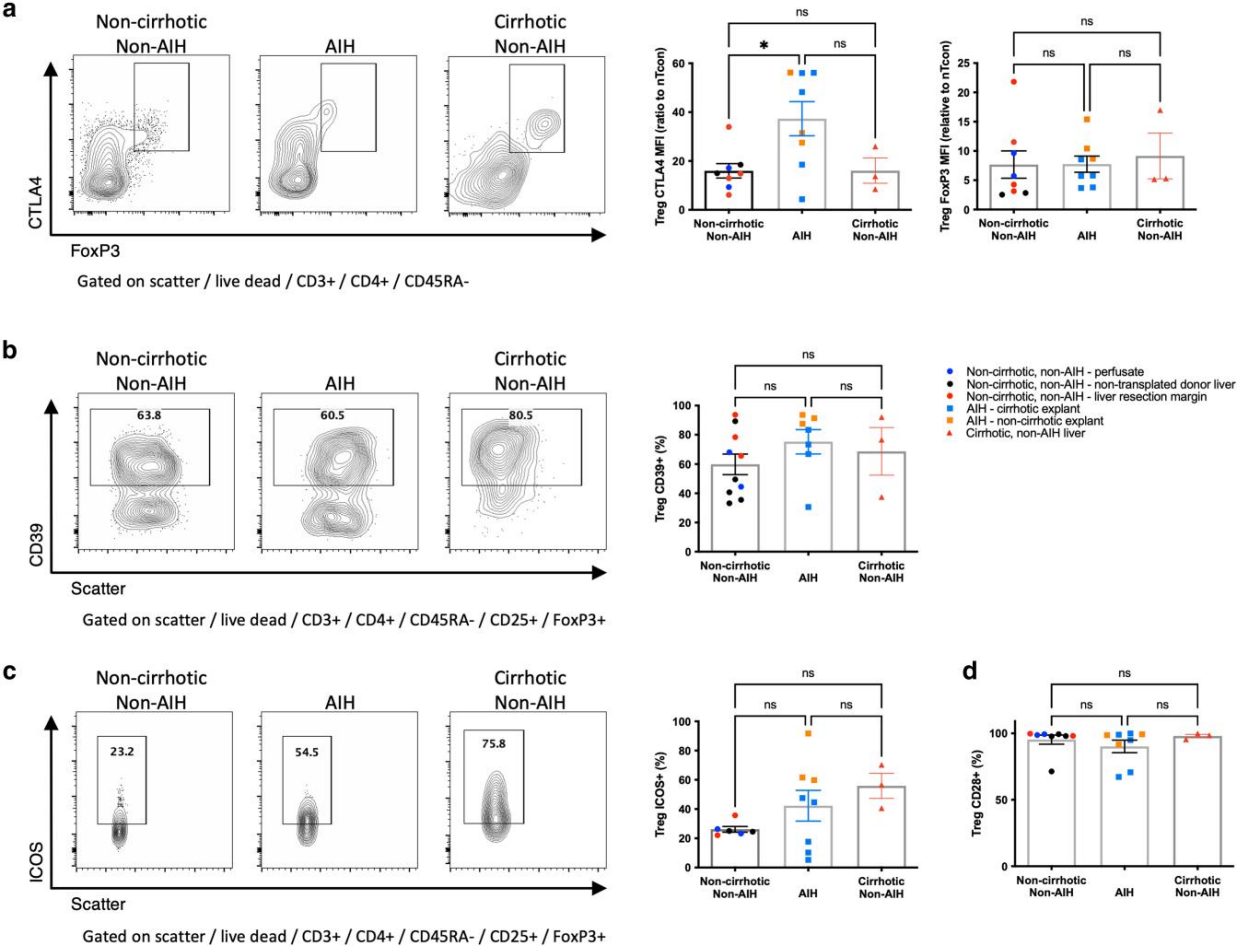
Comparison of the phenotype of CD4 + CD45RA-CD25 + FoxP3+ Treg from liver tissue. Comparison of a: CTLA4 expression between liver tissue from patients with AIH to those without. b: CD39 expression c: ICOS expression d: CD28 expression. Flow cytometry plots show a representative sample from each group, graphs show aggregate data for all samples tested with MFI normalized to an internal negative population (naive Tcon (CD3 + CD4 + CD45RA + FoxP3−)) where stated. ns, non-significant, * *P* < 0.05, by one way ANOVA with Tukey’s multiple comparison test.

We next assessed the phenotype of Treg from AIH, non-AIH noncirrhotic, and non-AIH cirrhotic liver. We observed significantly higher CTLA4 expression in AIH compared with non-AIH non-cirrhotic or cirrhotic liver [CTLA4 average MFI (ratio to CTLA4 MFI of naïve Tcon), 37.31, 15.96, and 16.05, respectively, *P* < 0.05] (Fig. 2a). Otherwise, expressions of other functional markers, including FoxP3, CD39, ICOS, and CD25 (Figs 1b and 2a–c), were broadly similar. Likewise, CD28 expression was maintained (Fig. 2d), suggesting that despite the high expression of CTLA4, potential costimulation via CD28 may still be possible. Further subanalysis, separating (sub)acute AIH from cirrhotic AIH, showed similar trends, although with a trend to greater FoxP3, CD39, and ICOS expression in the acute setting and a trend of reduced CD28 in Treg in cirrhotic AIH (Supplementary Figure S3).

**Figure 3:**
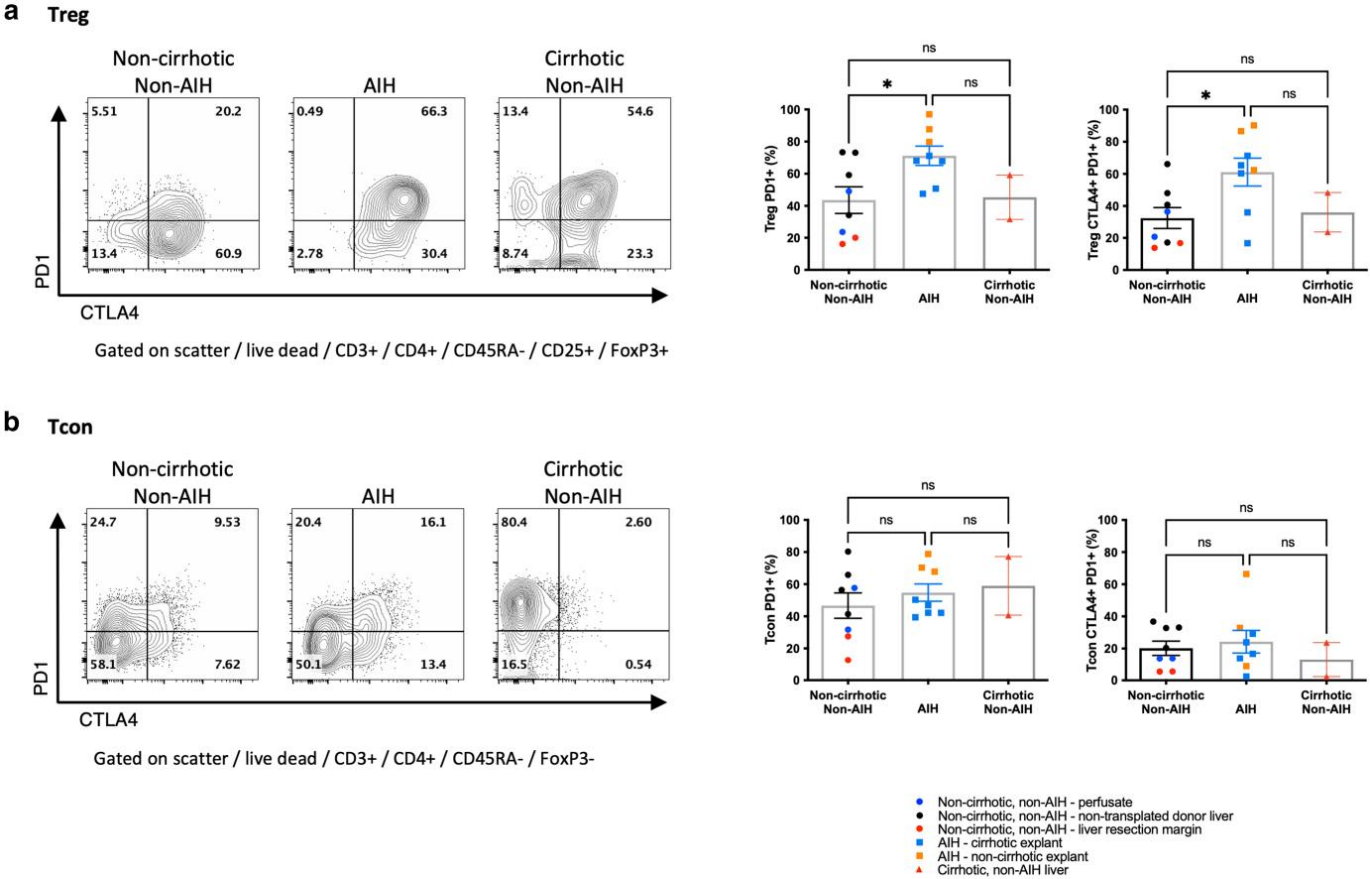
Comparison of Treg and Tcon CTLA4 and PD1 expression in liver tissue. a: CD4 + CD45RA-CD25 + FoxP3+ Treg and b: CD4 + CD45RA-FoxP3− memory Tcon expression of PD1 and the frequency of CTLA4 + PD1+ subsets, comparing AIH to noncirrhotic, non-AIH, or non-AIH cirrhotic liver. Flow cytometry plots show a representative sample from each group, graphs show aggregate data for all samples tested. ns, non-significant, * *P* < 0.05, by one way ANOVA with Tukey’s multiple comparison test.

PD1 expression was enhanced in Treg from AIH livers compared with non-AIH noncirrhotic and cirrhotic liver; on average, 71.2% of Treg expressed PD1 vs 43.6% and 45.4%, respectively (*P* < 0.05). This suggested that in AIH, the majority of Treg express PD1 and CTLA4 to a higher level compared with non-AIH settings (Fig. 3a). This observation was specific to Treg and not seen in Tcon populations from the same liver samples (Fig. 3b). The principle elevation in Treg PD1 was seen in those with active inflammation, compared with less active, cirrhotic AIH (Supplementary Figure S4).

### In AIH, hepatic Tcon had elevated HLA-DR expression

**Figure 4:**
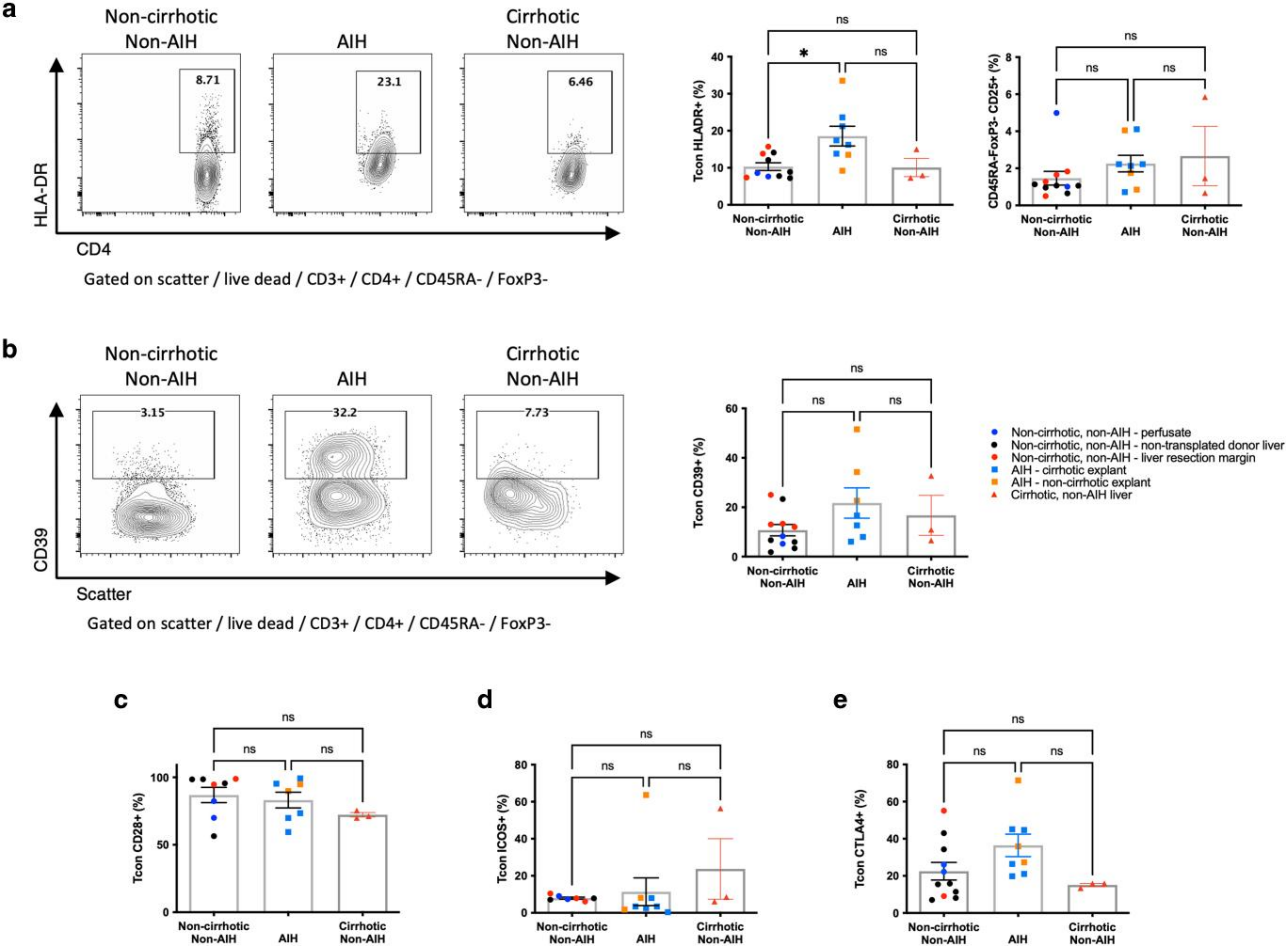
Comparison of CD4 + CD45RA-FoxP3− memory Tcon activation and phenotype between AIH liver and noncirrhotic, non-AIH, and cirrhotic liver. Frequency of a: HLA-DR expression b: CD39, c: CD28, d: ICOS, and e: CTLA4 expression. Flow cytometry plots show a representative sample from each group, graphs show aggregate data for all samples tested. ns, non-significant, * *P* < 0.05, by one way ANOVA with Tukey’s multiple comparison test.

We next tested whether Tcon had differences in phenotype comparing AIH and non-AIH liver. We observed a significant increase in HLA-DR expression in AIH compared with non-AIH noncirrhotic and cirrhotic Tcon (mean: 18.6%, 10.3%, and 10.1%, respectively, *P* < 0.05) (Fig. 4a), suggesting chronic Tcon activation in AIH. Notably, this was observed in the AIH cirrhosis patients undergoing transplantation for chronic liver disease, all but one of whom met conventional complete biochemical response criteria and had minimal inflammatory activity on histological examination (Table 1). While there was a trend toward increased CTLA4 and CD39 in the AIH group, which may suggest evidence of recent activation, no significant differences in CD39, CD28, ICOS, CTLA4, or PD1 expression in Tcon were observed (Figs 3b and 4a–e). Subanalysis, separating (sub)acute AIH from cirrhotic AIH, showed similar trends (Supplementary Figure S5), and in acute AIH, Tcon more frequently expressed CD39, with a trend increasing for CTLA4, again likely representing acute activation.

### CD28 ligand availability on circulating immune cells isolated from the liver

**Figure 5:**
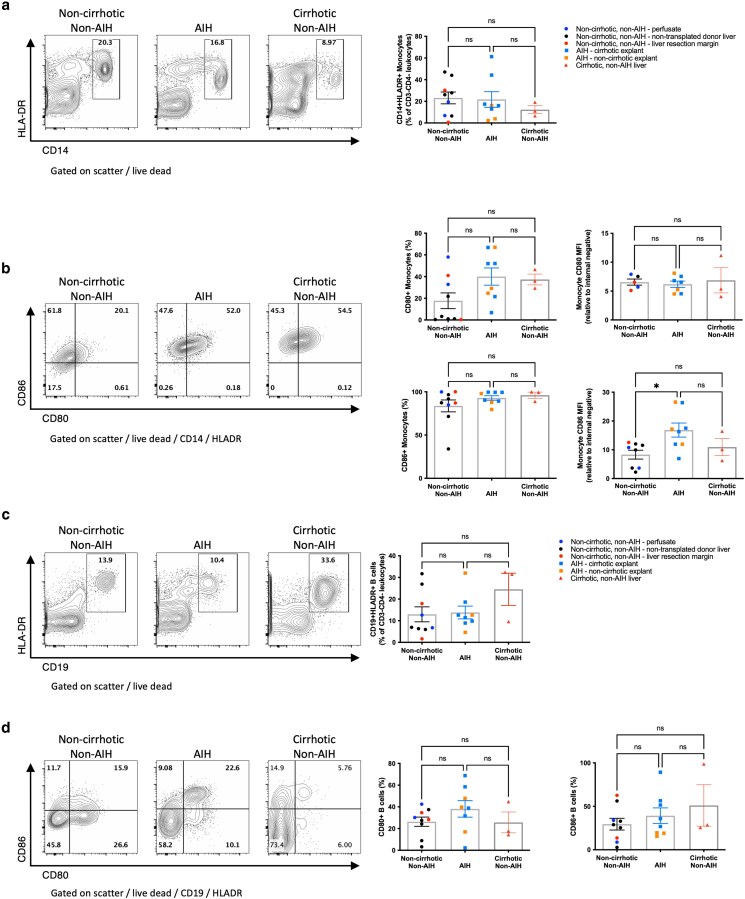
Comparison of intrahepatic monocyte and B cell populations between AIH liver and noncirrhotic, non-AIH, and cirrhotic liver. a: frequency CD14 + HLA-DR+ monocytes. b: CD80 and CD86 expression by monocytes demonstrating maintained frequency and intensity of CD80 expression and maintained frequency and increased intensity of CD86 expression by monocytes in AIH liver. c: Frequency CD19 + HLA-DR + B cells; b: CD80 and CD86 expression in B cells demonstrating maintained frequency of CD80 and CD86 expression by B cells in the liver in AIH. Flow cytometry plots show a representative sample from each group, graphs show aggregate data for all samples tested with MFI normalized to an internal negative population (CD80/CD86-negative CD3+ T lymphocytes) where stated. ns, non-significant, * *P* < 0.05, by one way ANOVA with Tukey’s multiple comparison test.

As we had observed an increase in Treg frequency and expression of CTLA4, which have a major role in the control of availability of CD80 and CD86, and evidence of ongoing Tcon stimulation in AIH, we next explored whether there were differences in the availability of CD80 and CD86 on monocytes and B cells that had been exposed to the liver microenvironment. We observed that CD14 + HLADR+ monocyte frequencies were similar in AIH and non-AIH liver samples (Fig. 5a). However, CD80, which is typically absent or minimally expressed in peripheral blood monocytes [24], was commonly observed on hepatic monocytes with a trend to a greater frequency of CD80-positive monocytes in AIH compared with non-AIH, noncirrhotic liver, suggesting differentiation and activation in tissues that was more pronounced in AIH. As expected, CD86 expression was near universal in monocytes, but with significantly higher MFI in AIH compared with non-AIH liver (normalized against internal negative population MFI) (Fig. 5b). Subanalysis, separating (sub)acute AIH from cirrhotic AIH, showed similar trends (Supplementary Figure S6a–e).

Similar to monocytes, B cell frequencies in AIH and non-AIH liver were similar, and B cells had preserved levels of CD80 and CD86 in AIH compared with non-AIH tissue (Fig. 5c and d), again with similar trends when subgrouped by (sub)acute and chronic AIH (Supplementary Figure S6f–h). Overall, these observations implied that there was a relative excess of Treg and the effector molecules capable of controlling CD80 and CD86 in AIH, but nonetheless, CD80 and CD86 levels were maintained, suggesting a possible imbalance in Treg CD80 and CD86 control and inflammatory drivers of CD80 and CD86.

### Intrahepatic Treg from patients with AIH remained functionally capable of CD80 transendocytosis

**Figure 6:**
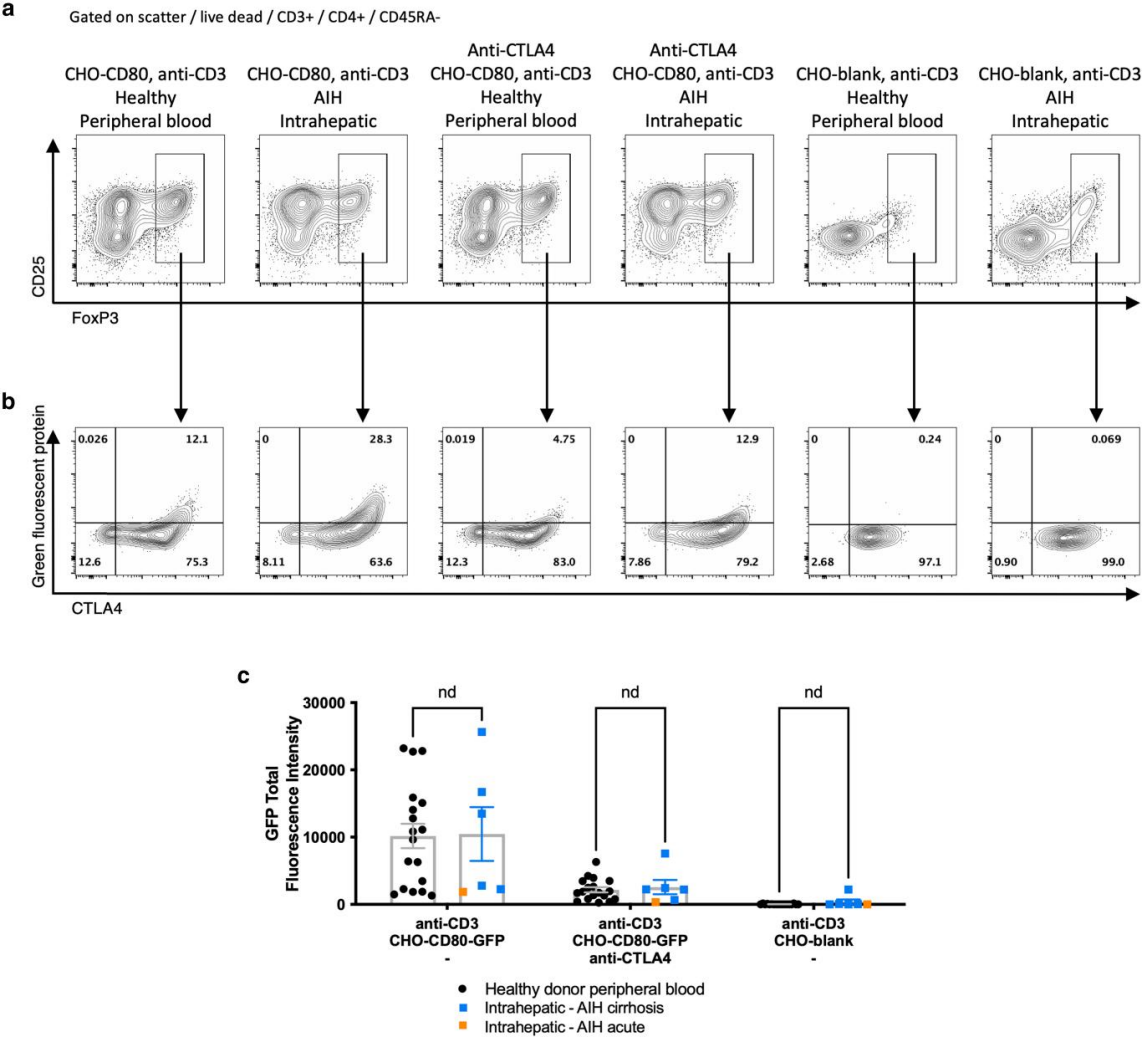
Functional assessment of CD80 transendocytosis by Treg comparing intrahepatic Treg from patients with AIH to peripheral blood Treg from healthy donors. a: Total memory CD4 T cell expression of FoxP3 and CD25 after 16 hours stimulation *in vitro* with anti-CD3 antibody with and without CD80 and with and without blocking anti-CTLA4 antibody b: representative flow cytometry plots from each set of conditions, demonstrating CD80-green fluorescence protein (GFP) uptake, as a marker of transendocytosis, and CTLA4 expression within FoxP3+ Treg Treg after 16 hours stimulation *in vitro*. c: Total fluorescence intensity of GFP in CD4 + CD45RA-CD25 + FoxP3+ Treg after 16 hours stimulation, reflecting acquisition of CD80-GFP from donor CHO cells. Flow cytometry plots show a representative sample from each group, graphs show aggregate data for all 18 peripheral blood samples and 6 liver samples tested. nd, no difference by multiple independent T tests.

As we had demonstrated continued CD80 and CD86 availability in AIH despite high levels of phenotypically active Treg, we tested whether the Treg had the capacity to transendocytose CD80 from model APCs, a critical strategy for CD80 control. In our assay, acquisition of GFP-labelled CD80 from CHO cells by FoxP3+ Treg was assessed. We observed that Treg from AIH liver were readily stimulated with CD80 and soluble anti-CD3, upregulating CD25 expression, suggesting that even in the presence of high CTLA4 expression, they remained responsive to CD28 costimulation (Fig. 6a). Hepatic Treg from AIH liver tissue acquired GFP-CD80 at a level similar to healthy peripheral blood Treg (total GFP fluorescence intensity: 10 173 vs 10 458, *P* = 0.94) (Fig. 6b and c). This acquisition was suppressed by blocking with anti-CTLA4 antibody and abrogated in the absence of CD80, demonstrating the specificity of the assay (Fig. 6b and c).

### CD80 transendocytosis was enhanced by IL10 or IL2

**Figure 7:**
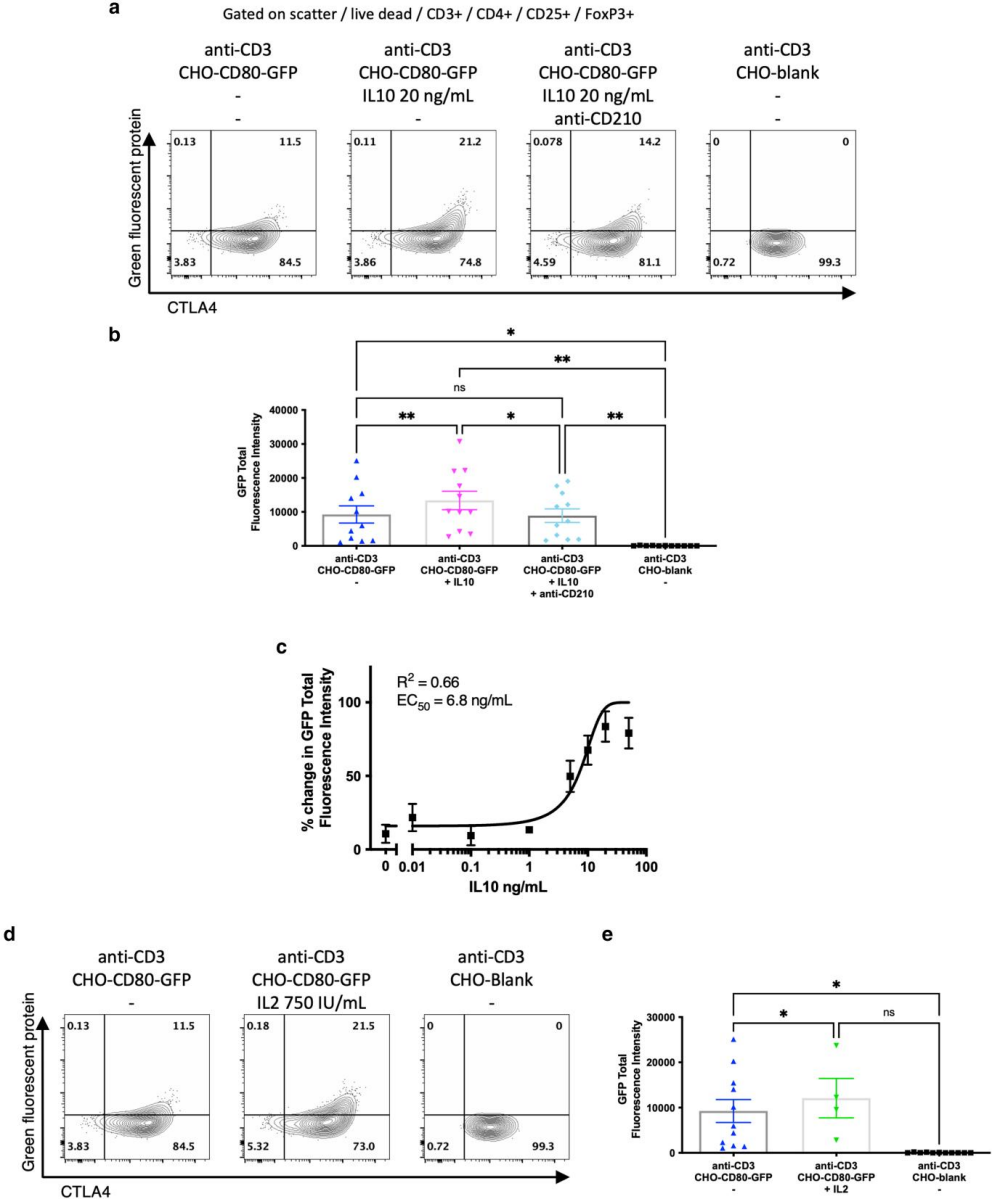
Effect of IL10 and IL2 on CD80 transendocytosis by peripheral blood CD3 + CD4 + CD25 + FoxP3+ Treg from healthy donors. a: Representative flow cytometry data demonstrating green fluorescent protein (GFP)-labelled CD80 acquisition by transendocytosis in Treg after 16 hours stimulation *in vitro* with and without 20 ng/ml IL10, 20 ng/ml IL10 and 20 μg/ml anti-CD210 (IL10 receptor blocking antibody) and in the absence of CD80 as GFP intensity against total cell CTLA4, as stated. b: Aggregate data from 11 independent experiments. c: Dose response curve of IL10 concentration and total CD80-GFP acquisition by Treg after 16 hours stimulation. d: Representative flow cytometry data, and e: Aggregate data from at least four independent experiments demonstrating CD80-GFP uptake in Treg after 16 hours, similarly to (a) but comparing with and without 750 IU/ml IL2. ns, non-significant, * *P* < 0.05, ** *P* < 0.01 by one way ANOVA with Tukey’s multiple comparison test.

We had observed preserved functional capacity for CD80 transendocytosis in hepatic Treg from patients with AIH. Impaired IL10 production and IL2 availability in the liver have been demonstrated in AIH [4, 25], and in murine models, an IL2-mutein increases Treg transendocytosis of CD80 and CD86 [26]. Therefore, we next tested whether CD80 transendocytosis activity could be modulated by these cytokines. We observed that peripheral blood Treg from healthy donors had significant increases in CD80-GFP acquisition from model APCs with the addition of 20 ng/ml IL10 (TFI 9253 vs 13,368, *P* < 0.01), which was abrogated by the presence of blocking anti-IL10 receptor (anti-CD210) antibody (Fig. 7a and b). IL10’s effect on TE was dose-dependent with an EC50 of 6.8 ng/ml (Fig. 7c). Similarly, addition of 750 IU IL2 was associated with a significant increase in CD80-GFP acquisition (TFI 9253 vs 12 087, *P* < 0.05) by Treg from model APCs (Fig. 7d and e).

## Discussion

Treg are critical for the maintenance of self-tolerance, and their dysfunction or depletion has been implicated in many autoimmune conditions, including AIH. However, data regarding Treg frequency in AIH are contradictory; they differ with treatment, disease stage, degree of inflammation, and potentially ethnicity, and reports have been based on peripheral blood analyses [4–7, 27–30]. Assessment of hepatic Treg in AIH by immunohistology techniques has demonstrated increased Treg frequency in AIH, which falls with immunosuppressive treatment and treatment response [27, 31–33]. Notably, changes in intrahepatic Treg frequency and phenotype are not reflected in peripheral blood analyses [32]; hence, it is important to study liver-derived Treg directly. Functional defects in peripheral blood Treg have been reported in AIH, including defective IL10 production, impaired IL2 responses, and defective adenosine production through impaired upregulation of CD39, an ectonucleotidase involved in the production of immunosuppressive adenosine [4, 6, 28, 34]. However, dissecting the impact of pharmacological treatment on Treg from inherent Treg (dys)function and understanding whether these peripheral blood Treg changes are relevant in the liver environment remains a challenge. Here, we report the phenotype of intrahepatic Treg and Tcon in advanced stages of AIH with (sub)acute liver failure or cirrhosis when patients were undergoing liver transplantation, compared with non-AIH liver both with and without underlying liver disease. We also demonstrate functional assessment of Treg from the intrahepatic compartment in AIH, with respect to their capacity for CD80 transendocytosis.

In the advanced AIH liver tissue assessed, Treg were increased as a proportion of the total lymphocyte pool compared with other liver tissue settings, with trends to increase in both acute and chronic AIH, implying that a paucity of Treg in the liver microenvironment is not an obvious feature of AIH. This is consistent with a prior study reporting greater frequencies of FoxP3+ Treg from the liver of patients with AIH and other inflammatory conditions, compared with normal liver tissue [35]. In our study, Treg frequencies were maintained irrespective of the presence of histological or biochemical inflammatory activity. It is unlikely this was due to changes associated with cirrhosis, as it was not observed in cirrhotic alcohol–related liver disease tissue, and a similar pattern was seen in actively inflamed noncirrhotic AIH (acute disease) and cirrhotic AIH. These observations suggest that in advanced stages, AIH is not characterized by a numerical deficiency of Treg nor a failure of Treg to home to the liver.

The phenotype of Treg from cirrhotic or (sub)acute liver failure liver tissue with AIH differed from that of noncirrhotic, non-AIH liver tissue and cirrhotic, non-AIH liver tissues. We observed a significant increase in Treg CTLA4 and PD1 in hepatic Treg in AIH compared with both cirrhotic and noncirrhotic non-AIH liver. This suggests that the observed expression of PD1 and CTLA4 in peripheral blood Treg in AIH [8] is both present at the site of active inflammation and significantly different from non-AIH contexts. Furthermore, our data suggest that the PD1 + CTLA4 high Treg phenotype persists in the liver, despite the proposed reduction in PD1 expression observed following immunosuppressive treatment in peripheral blood [8]. We observed that Treg display a broadly activated memory Treg phenotype with preserved expression of ICOS, CD39, CD25, FoxP3, and CD28 and preserved functional activity with the capacity to activate and undertake CD80 transendocytosis, strongly implying that they are not exhausted. PD1 expression is known to enhance the development, homeostasis, and function of Treg [36, 37], suggesting a critical role for PD1 in competent Treg function. The maintained expression of CD39 in hepatic Treg in AIH suggested that although previously shown to be low in peripheral blood [6], populations of these cells are preserved in the liver microenvironment in AIH. However, their reduction in the periphery and failure to adequately control Th17 cells [6] may still be important in the initiation or potentiation of autoreactive immune responses outside of the liver.

The capacity of Treg freshly isolated from tissue to perform transendocytosis of CD80 from a model APC represents a useful, reproducible measure of Treg stimulation and one axis of Treg function [38, 39]. It allows *ex vivo* assessment of cells isolated from different disease states and reflects one of the critical effector mechanisms that are employed by Treg in maintaining tolerance [18].

CTLA4 is strongly implicated in the maintenance of self-tolerance in the liver. CTLA4 is a homolog of CD28, and both receptors share the same two ligands, CD80 and CD86, although CTLA4 binds both with higher affinity than CD28. CTLA4 negatively regulates CD28 function by competing with CD28 to bind the ligands and by physical removal of CD80 and CD86 from APCs, reducing the costimulation signals available to T cells [40]. Genetic studies have demonstrated an association between AIH and an enhancer variant that reduces the expression of CTLA4 [3], and targeted gene studies support associations between CTLA4 variants and AIH [41–43]. Furthermore, both treatment with anti-CTLA4 immunotherapy for cancer and CTLA4 haploinsufficiency are associated with inflammatory liver complications [19, 44], demonstrating the importance of CTLA4 in immune control in the liver. Further evidence of the importance of this regulatory locus is seen with a murine model of CD28 agonism resulting in an immune-mediated hepatitis [20]. Therefore, the balance of CTLA4 and the CD28 costimulation pathway appears to be critical in the regulation of immune tolerance in the liver. Treg in the liver are found in close physical association with dendritic cells [35], one of several APCs in the liver that express CD80 and CD86, and are appropriately localized in tissues.

We observed that immune cells circulating to the liver, which typically express CD80 and CD86, had at least maintained or increased expression of CD80 and CD86 in these states of advanced AIH disease. Due to the presence of a higher proportion of Treg with greater CTLA4 expression, it might be expected that these levels would be lower, and this observation suggests a possible imbalance between the availability of CD80 and CD86 and Treg function. A similar observation in another autoimmune liver disease, primary biliary cholangitis (PBC), where greater hepatic APC expression of CD80 and CD86 was reported [45], suggests that this may be a feature of hepatic autoimmunity. In future work, the factors regulating CD80 and CD86 should be explored in autoimmune hepatitis, as the upregulation of CD80 and CD86 is part of the inflammatory response to enable T cell costimulation [46]. Why CD80 and CD86 levels are preserved or relatively elevated could be related to genetic variants [47, 48], the treatments received by patients, the result of chronic inflammatory stimulation, and liver-specific expression (CD80 and CD86 are expressed and dynamically regulated by IL10 in liver-specific cells such as liver sinusoidal endothelial cells [15, 49, 50]). Elevated CD86 expression in PBC was inversely associated with Treg frequency, and *in vitro* Treg were less capable of controlling CD86 levels [45]. Although we demonstrated preserved Treg transendocytosis capacity, these data support the concept of Treg control of costimulation being critical in the regulation of immune responses in the human liver. However, the pathogenesis of AIH is likely multifactorial, and the observed imbalance of CD80 and CD86 availability and Treg activity and ligand control is likely one aspect of a complex series of immunological and other factors resulting in disease.

Our study demonstrated nonexhausted, active Treg, which were competent at CD80 transendocytosis, but a relative excess of CD80 and CD86 in AIH; therefore, we studied the potential impact of environmental cytokines on Treg transendocytosis activity. In AIH, levels of IL2 in the liver are low, and Treg have impaired responses to IL2, and low levels of IL10 are secreted [4, 25]. We demonstrated that CD80 transendocytosis can be increased following treatment with IL2 or IL10, which suggests a potential mechanism that may either underlie the imbalance between Treg frequency and phenotype compared with CD80 and CD86 availability or may serve as potential therapeutic avenues. Although we have not demonstrated that there is aberrant signalling in the IL2 or IL10 pathways in the liver in AIH there is interest in therapeutic manipulation of these pathways. Low-dose IL2 treatment selectively enhances Treg phenotype and function in autoimmune liver diseases [51, 52] hence have efficacy in the management of AIH [53]. Interestingly, a role for IL2 in upregulating transendocytosis by Treg illustrates the important interactions between the CD25-IL2 pathway and the regulation of CD80 and CD86 and IL10. Recent data have shown that long-lived potent IL2 muteins can enhance Treg transendocytosis *in vivo* and impair dendritic cell maturation by the downregulation of class II MHC via an IL10-dependent pathway [26]. These observations suggest complex proregulatory interactions between the CTLA4, IL2, and IL10 pathways, which may have the potential to restore control of the activated, proinflammatory environment seen in the liver in AIH.

We demonstrated that hepatic Tcon in AIH had evidence of recent, chronic activation with high levels of HLA-DR expression and trends towards more CD25, CD39, and CTLA4 expression, particularly in the acute setting. However, these trends were observed even in patients meeting histological remission and biochemical response criteria, demonstrated by the absence of significant inflammatory infiltrate on histological assessment and normal ALT and IgG [23]. Others have previously reported that ongoing inflammatory activity can be identified in patients reaching conventional biochemical treatment response criteria and that this is associated with progressive disease and elevated mortality risk [54]. Furthermore, alterations in circulating immune cell populations in AIH are not necessarily restored with conventional treatments, despite achieving biochemical response [55], implying persistent immunological changes in AIH. These observations imply that immunological activity persists despite patients meeting conventional treatment response criteria. Therefore, improved biomarkers of treatment response, including intrahepatic immune parameters, should be sought with the aim of defining deep immunological remission and testing whether this is associated with differences in treatment outcome.

This study is not an exhaustive characterization of the intrahepatic immune microenvironment in AIH due to the small amounts of tissue available. We focused on specific markers relevant to the CD28 axis in CD4 T cells; hence, wider perturbations of Treg, CD4, CD8, and other immune cell subsets may exist. Additionally, due to the destructive nature of immune cell isolation from these samples, we were unable to assess the phenotype and expression of costimulatory and other proteins in liver-specific cells, such as hepatocytes, LSEC, Kupffer cells, and hepatic dendritic cells. However, the observations here suggest that there is an imbalance in the control of the CD28 pathway in AIH that warrants further exploration as both a route to understanding disease pathogenesis and potential druggable treatment targets. The tissue available for study was for patients undergoing liver transplantation and therefore represents an advanced stage of AIH. Therefore, it is possible differing immune phenotypes and dysregulation may occur at the initiation of disease, in earlier phases or in milder disease.

We did not explicitly demonstrate tissue residency of the Treg assessed in these experiments, and some of the isolated Treg may represent transient cells from the hepatic circulation. However, due to the flushing and rinsing of samples this is likely to be a minority of the cells. Tissue-resident Treg, typically defined by markers such as CD69 and CD103, show a similar range of transcriptional profiles across different nonlymphoid organs, and there is evidence for transience of tissue residency [56], suggesting that disease-specific Treg changes are either induced *in situ* at the site of disease or represent a generalized Treg difference in patients. Within the broad group of CD25 + FoxP3+ Treg, significant heterogeneity exists from circulating vs tissue-resident Treg, thymic vs peripherally derived Treg, and differing functional states such as naive, effector, central memory, and effector memory Treg [57]. Future work will be required to explore specific subsets of tissue Treg in AIH.

In this study, we compared the phenotype of intrahepatic T cells from patients with AIH (both cirrhotic and noncirrhotic) with two control groups. Noncirrhotic, non-AIH liver tissue represents the absence of both features, which could influence phenotype, and cirrhotic, non-AIH to account for the influence of cirrhosis. The noncirrhotic, non-AIH liver tissue was derived from sources that may not represent truly healthy liver, including deceased donors (perfusate or donated but nontransplanted liver) or from patients often with malignancy (surgical resection margins). However, no consistent differences were observed between the tissue sources, suggesting comparable phenotypes. The use of alcohol-related cirrhosis as a cirrhotic control was selected as the patients were abstinent from alcohol use to be eligible for transplantation; hence, the influence on immune cell phenotype from alcohol consumption should be minimized. A further caution in the interpretation of these data is that there is interindividual variability in some observations, and the number of samples in the study is small. Although variation may be expected due to the variation in disease activity, course, and stage between individuals, the overall observations are suggestive of patterns of difference in the phenotype of immune cells in the liver in AIH.

There is an unmet need for new biomarkers to risk-stratify patients with AIH and to identify predictors of incomplete response to treatment (both conventional and novel immunological markers) to allow treatment intensification in those who require it and avoidance of excess treatment in those who do not. Approximately 20% of patients have inadequate response or intolerance to currently available treatments, and in real-world studies >40% of patients are not in remission, despite pharmacotherapy [58], illustrating the need for better monitoring and therapeutics. Understanding the immunopathogenesis of AIH may identify potential new treatment targets, such as the CD28 pathway, new measures of remission, and prognostic markers, thereby allowing personalization of treatment for patients with AIH.

In summary, we have demonstrated that in hepatic tissue from patients with advanced AIH, there is a relative excess of Treg, with a PD1, CTLA4 bright phenotype. These Treg are functional, stimulate readily, and can perform CD80 transendocytosis in a model system, implying that they have the capacity to competently regulate the CD28 costimulation axis. However, we observed an excess of CD80 and CD86 on immune cells in the liver, implying a functional imbalance in the control of costimulatory ligands. This suggests possible mechanistic and therapeutic targets to be explored, including targeting of CD80 and CD86, CD28 signalling, and enhancing Treg function to improve control of costimulation ligand availability. However, AIH has multiple pathogenic mechanisms, and these observations likely represent one of many contributing pathways.

## Supplementary Material

uxag013_Supplementary_Data

## Data Availability

Individual data are available by request to the corresponding author.

## References

[1] Heneghan MA, Yeoman AD, Verma S, Smith AD, Longhi MS. Autoimmune hepatitis. Lancet (London, England) 2013, 382, 1433–44. 23768844 10.1016/S0140-6736(12)62163-1

[2] de Boer YS, van Gerven NMF, Zwiers A, Verwer BJ, van Hoek B, van Erpecum KJ, et al Genome-wide association study identifies variants associated with autoimmune hepatitis type 1. Gastroenterology 2014, 147, 443–52.e5. 24768677 10.1053/j.gastro.2014.04.022

[3] Li Y, Sun Y, Liu Y, Wang B, Li J, Wang H, et al Genome-wide meta-analysis identifies susceptibility loci for autoimmune hepatitis type 1. Hepatology 2022, 76, 564–75. 35184318 10.1002/hep.32417

[4] Liberal R, Grant CR, Holder BS, Cardone J, Martinez-Llordella M, Ma Y, et al In autoimmune hepatitis type 1 or the autoimmune hepatitis-sclerosing cholangitis variant defective regulatory T-cell responsiveness to IL-2 results in low IL-10 production and impaired suppression. Hepatology 2015, 62, 863–75. 25953611 10.1002/hep.27884

[5] Longhi MS, Ma Y, Bogdanos DP, Cheeseman P, Mieli-Vergani G, Vergani D. Impairment of CD4(+)CD25(+) regulatory T-cells in autoimmune liver disease. J Hepatol 2004, 41, 31–7. 15246204 10.1016/j.jhep.2004.03.008

[6] Grant CR, Liberal R, Holder BS, Cardone J, Ma Y, Robson SC, et al Dysfunctional CD39(POS) regulatory T cells and aberrant control of T-helper type 17 cells in autoimmune hepatitis. Hepatology 2014, 59, 1007–15. 23787765 10.1002/hep.26583 PMC6377365

[7] Liu Y, Yan W, Yuan W, Wang P, Huang D, Luo X, et al Treg/Th17 imbalance is associated with poor autoimmune hepatitis prognosis. Clinical Immunology 2019, 198, 79–88. 30453094 10.1016/j.clim.2018.11.003

[8] Jeffery HC, Braitch MK, Bagnall C, Hodson J, Jeffery LE, Wawman RE, et al Changes in natural killer cells and exhausted memory regulatory T cells with corticosteroid therapy in acute autoimmune hepatitis. Hepatol Commun 2018, 2, 421–36. 29619420 10.1002/hep4.1163 PMC5880196

[9] Fontenot JD, Gavin MA, Rudensky AY. Foxp3 programs the development and function of CD4+ CD25+ regulatory T cells. Nat Immunol 2003, 4, 330–6. 12612578 10.1038/ni904

[10] Sakaguchi S, Sakaguchi N, Asano M, Itoh M, Toda M. Immunologic self-tolerance maintained by activated T cells expressing IL-2 receptor alpha-chains (CD25). Breakdown of a single mechanism of self-tolerance causes various autoimmune diseases. J Immunol 1995, 155, 1151–64. 7636184

[11] Yilmaz K, Haeberle S, Kim YO, Fritzler MJ, Weng SY, Goeppert B, et al Regulatory T-cell deficiency leads to features of autoimmune liver disease overlap syndrome in scurfy mice. Front Immunol 2023, 14, 1253649. 37818371 10.3389/fimmu.2023.1253649 PMC10561387

[12] Lopez SI, Ciocca M, Oleastro M, Cuarterolo ML, Rocca A, de Dávila MT, et al Autoimmune hepatitis type 2 in a child with IPEX syndrome. J Pediatr Gastroenterol Nutr 2011, 53, 690–3. 21629128 10.1097/MPG.0b013e3182250651

[13] Barzaghi F, Amaya Hernandez LC, Neven B, Ricci S, Kucuk ZY, Bleesing JJ, et al Long-term follow-up of IPEX syndrome patients after different therapeutic strategies: an international multicenter retrospective study. J Allergy Clin Immunol 2018, 141, 1036–49.e5. 29241729 10.1016/j.jaci.2017.10.041 PMC6050203

[14] Esensten JH, Helou YA, Chopra G, Weiss A, Bluestone JA. CD28 costimulation: from mechanism to therapy. Immunity 2016, 44, 973–88. 27192564 10.1016/j.immuni.2016.04.020 PMC4932896

[15] Diehl L, Schurich A, Grochtmann R, Hegenbarth S, Chen L, Knolle PA. Tolerogenic maturation of liver sinusoidal endothelial cells promotes B7-homolog 1-dependent CD8+ T cell tolerance. Hepatology 2008, 47, 296–305. 17975811 10.1002/hep.21965

[16] Herkel J, Jagemann B, Wiegard C, Lazaro JF, Lueth S, Kanzler S, et al MHC class II-expressing hepatocytes function as antigen-presenting cells and activate specific CD4 T lymphocyutes. Hepatology 2003, 37, 1079–85. 12717388 10.1053/jhep.2003.50191

[17] Gao D, Li J, Orosz CG, Bumgardner GL. Different costimulation signals used by CD4(+) and CD8(+) cells that independently initiate rejection of allogenic hepatocytes in mice. Hepatology 2000, 32, 1018–28. 11050052 10.1053/jhep.2000.19325

[18] Qureshi OS, Zheng Y, Nakamura K, Attridge K, Manzotti C, Schmidt EM, et al Trans-endocytosis of CD80 and CD86: a molecular basis for the cell-extrinsic function of CTLA-4. Science (New York, NY) 2011, 332, 600–3. 10.1126/science.1202947 PMC3198051 21474713

[19] Schubert D, Bode C, Kenefeck R, Hou TZ, Wing JB, Kennedy A, et al Autosomal dominant immune dysregulation syndrome in humans with CTLA4 mutations. Nat Med 2014, 20, 1410–6. 25329329 10.1038/nm.3746 PMC4668597

[20] Corse E, Gottschalk RA, Park JS, Sepulveda MA, Loke P, Sullivan TJ, et al Cutting edge: chronic inflammatory liver disease in mice expressing a CD28-specific ligand. J Immunol 2013, 190, 526–30. 23248264 10.4049/jimmunol.1202621 PMC4964790

[21] Hennes EM, Zeniya M, Czaja AJ, Parés A, Dalekos GN, Krawitt EL, et al Simplified criteria for the diagnosis of autoimmune hepatitis. Hepatology 2008, 48, 169–76. 18537184 10.1002/hep.22322

[22] Jonsson JR, Hogan PG, Balderson GA, Ooi LL, Lynch SV, Strong RW, et al Human liver transplant perfusate: an abundant source of donor liver-associated leukocytes. Hepatology 1997, 26, 1111–4. 9362349 10.1002/hep.510260504

[23] Pape S, Snijders R, Gevers TJG, Chazouilleres O, Dalekos GN, Hirschfield GM, et al Systematic review of response criteria and endpoints in autoimmune hepatitis by the international autoimmune hepatitis group. J Hepatol 2022, 76, 841–9. 35066089 10.1016/j.jhep.2021.12.041

[24] Boyette LB, Macedo C, Hadi K, Elinoff BD, Walters JT, Ramaswami B, et al Phenotype, function, and differentiation potential of human monocyte subsets. PLoS One 2017, 12, e0176460. 28445506 10.1371/journal.pone.0176460 PMC5406034

[25] Chen YY, Jeffery HC, Hunter S, Bhogal R, Birtwistle J, Braitch MK, et al Human intrahepatic regulatory T cells are functional, require IL-2 from effector cells for survival, and are susceptible to Fas ligand-mediated apoptosis. Hepatology 2016, 64, 138–50. 26928938 10.1002/hep.28517 PMC4950043

[26] Jamison BL, Lawrance M, Wang CJ, DeBerg HA, Ziegler LJ, Sansom DM , et al An IL-2 mutein increases regulatory T cell suppression of dendritic cells via IL-10 and CTLA-4 to promote T cell anergy. Cell Rep 2024, 43, 114938. 10.1016/j.celrep.2024.114938 PMC11602548 39488830

[27] Peiseler M, Sebode M, Franke B, Wortmann F, Schwinge D, Quaas A, et al FOXP3+ regulatory T cells in autoimmune hepatitis are fully functional and not reduced in frequency. J Hepatol 2012, 57, 125–32. 22425700 10.1016/j.jhep.2012.02.029

[28] Okumura A, Ishikawa T, Sato S, Yamauchi T, Oshima H, Ohashi T, et al Deficiency of forkhead box P3 and cytotoxic T-lymphocyte-associated antigen-4 gene expressions and impaired suppressor function of CD4(+)CD25(+) T cells in patients with autoimmune hepatitis. Hepatol Res 2008, 38, 896–903. 18624718 10.1111/j.1872-034X.2008.00349.x

[29] Ferri S, Longhi MS, De Molo C, Lalanne C, Muratori P, Granito A, et al A multifaceted imbalance of T cells with regulatory function characterizes type 1 autoimmune hepatitis. Hepatology 2010, 52, 999–1007. 20683931 10.1002/hep.23792

[30] Longhi MS, Ma Y, Mitry RR, Bogdanos DP, Heneghan M, Cheeseman P, et al Effect of CD4+ CD25+ regulatory T-cells on CD8 T-cell function in patients with autoimmune hepatitis. J Autoimmun 2005, 25, 63–71. 10.1016/j.jaut.2005.05.001 16005184

[31] Diestelhorst J, Junge N, Schlue J, Falk CS, Manns MP, Baumann U, et al Pediatric autoimmune hepatitis shows a disproportionate decline of regulatory T cells in the liver and of IL-2 in the blood of patients undergoing therapy. PLoS One 2017, 12, e0181107. 28700730 10.1371/journal.pone.0181107 PMC5507441

[32] Taubert R, Hardtke-Wolenski M, Noyan F, Wilms A, Baumann AK, Schlue J, et al Intrahepatic regulatory T cells in autoimmune hepatitis are associated with treatment response and depleted with current therapies. J Hepatol 2014, 61, 1106–14. 24882050 10.1016/j.jhep.2014.05.034

[33] Derben FC, Ytting H, Hartleben B, Bantel H, Wedemeyer H, Willemoe GL, et al Salvage therapies of autoimmune hepatitis limit proinflammatory immune cells while sparing regulatory T cells. Hepatol Commun 2023, 7, e0088. 36976659 10.1097/HC9.0000000000000088 PMC10043582

[34] Vuerich M, Harshe R, Frank LA, Mukherjee S, Gromova B, Csizmadia E, et al Altered aryl-hydrocarbon-receptor signalling affects regulatory and effector cell immunity in autoimmune hepatitis. J Hepatol 2021, 74, 48–57. 32663496 10.1016/j.jhep.2020.06.044 PMC7749856

[35] Oo YH, Weston CJ, Lalor PF, Curbishley SM, Withers DR, Reynolds GM, et al Distinct roles for CCR4 and CXCR3 in the recruitment and positioning of regulatory T cells in the inflamed human liver. J Immunol 2010, 184, 2886–98. 20164417 10.4049/jimmunol.0901216

[36] Francisco LM, Salinas VH, Brown KE, Vanguri VK, Freeman GJ, Kuchroo VK, et al PD-L1 regulates the development, maintenance, and function of induced regulatory T cells. J Exp Med 2009, 206, 3015–29. 20008522 10.1084/jem.20090847 PMC2806460

[37] Amarnath S, Mangus CW, Wang JC, Wei F, He A, Kapoor V, et al The PDL1-PD1 axis converts human TH1 cells into regulatory T cells. Sci Transl Med 2011, 3, 111ra120. 10.1126/scitranslmed.3003130 PMC3235958 22133721

[38] Serwas NK, Hoeger B, Ardy RC, Stulz SV, Sui Z, Memaran N, et al Human DEF6 deficiency underlies an immunodeficiency syndrome with systemic autoimmunity and aberrant CTLA-4 homeostasis. Nat Commun 2019, 10, 3106. 31308374 10.1038/s41467-019-10812-x PMC6629652

[39] Davies EG, Cheung M, Gilmour K, Maimaris J, Curry J, Furmanski A, et al Thymus transplantation for complete DiGeorge syndrome: European experience. J Allergy Clin Immunol 2017, 140, 1660–70.e16. 28400115 10.1016/j.jaci.2017.03.020 PMC5716670

[40] Hou TZ, Qureshi OS, Wang CJ, Baker J, Young SP, Walker LS, et al A transendocytosis model of CTLA-4 function predicts its suppressive behavior on regulatory T cells. J Immunol 2015, 194, 2148–59. 25632005 10.4049/jimmunol.1401876 PMC4522736

[41] Agarwal K, Czaja AJ, Jones DE, Donaldson PT. Cytotoxic T lymphocyte antigen-4 (CTLA-4) gene polymorphisms and susceptibility to type 1 autoimmune hepatitis. Hepatology 2000, 31, 49–53. 10613727 10.1002/hep.510310110

[42] Djilali-Saiah I, Ouellette P, Caillat-Zucman S, Debray D, Kohn JI, Alvarez F. CTLA-4/CD 28 region polymorphisms in children from families with autoimmune hepatitis. Hum Immunol 2001, 62, 1356–62. 11756004 10.1016/s0198-8859(01)00344-5

[43] Eskandari-Nasab E, Tahmasebi A, Hashemi M. Meta-analysis: the relationship between CTLA-4+ 49 A/G polymorphism and primary biliary cirrhosis and type I autoimmune hepatitis. Immunol Invest 2015, 44, 331–48. 25942345 10.3109/08820139.2014.1003651

[44] Boutros C, Tarhini A, Routier E, Lambotte O, Ladurie FL, Carbonnel F, et al Safety profiles of anti-CTLA-4 and anti-PD-1 antibodies alone and in combination. Nat Rev Clin Oncol 2016, 13, 473–86. 27141885 10.1038/nrclinonc.2016.58

[45] Chen J, Hou X, Jia H, Cui G, Wu Z, Wang L, et al Regulatory T cells with a defect in inhibition on co-stimulation deteriorated primary biliary cholangitis. Oncotarget 2017, 8, 108406–17. 29312539 10.18632/oncotarget.22658 PMC5752452

[46] Sharpe AH, Freeman GJ. The B7-CD28 superfamily. Nat Rev Immunol 2002, 2, 116–26. 11910893 10.1038/nri727

[47] Dong M, Li J, Tang R, Zhu P, Qiu F, Wang C, et al Multiple genetic variants associated with primary biliary cirrhosis in a Han Chinese population. Clin Rev Allergy Immunol 2015, 48, 316–21. 25690649 10.1007/s12016-015-8472-0 PMC5584624

[48] Mells GF, Floyd JA, Morley KI, Cordell HJ, Franklin CS, Shin SY, et al Genome-wide association study identifies 12 new susceptibility loci for primary biliary cirrhosis. Nat Genet 2011, 43, 329–32. 21399635 10.1038/ng.789 PMC3071550

[49] Lohse AW, Knolle PA, Bilo K, Uhrig A, Waldmann C, Ibe M, et al Antigen-presenting function and B7 expression of murine sinusoidal endothelial cells and Kupffer cells. Gastroenterology 1996, 110, 1175–81. 8613007 10.1053/gast.1996.v110.pm8613007

[50] Knolle PA, Uhrig A, Hegenbarth S, Löser E, Schmitt E, Gerken G, et al IL-10 down-regulates T cell activation by antigen-presenting liver sinusoidal endothelial cells through decreased antigen uptake via the mannose receptor and lowered surface expression of accessory molecules. Clin Exp Immunol 2001, 114, 427–33. 10.1046/j.1365-2249.1998.00713.x PMC1905120 9844054

[51] Jeffery HC, Jeffery LE, Lutz P, Corrigan M, Webb GJ, Hirschfield GM, et al Low-dose interleukin-2 promotes STAT-5 phosphorylation, Treg survival and CTLA-4-dependent function in autoimmune liver diseases. Clin Exp Immunol 2017, 188, 394–411. 28176332 10.1111/cei.12940 PMC5422719

[52] Buitrago-Molina LE, Pietrek J, Noyan F, Schlue J, Manns MP, Wedemeyer H, et al Treg-specific IL-2 therapy can reestablish intrahepatic immune regulation in autoimmune hepatitis. J Autoimmun 2021, 117, 102591. 33387980 10.1016/j.jaut.2020.102591

[53] Lim TY, Martinez-Llordella M, Kodela E, Gray E, Heneghan MA, Sanchez-Fueyo A. Low-dose interleukin-2 for refractory autoimmune hepatitis. Hepatology 2018, 68, 1649–52. 29698571 10.1002/hep.30059

[54] Dhaliwal HK, Hoeroldt BS, Dube AK, McFarlane E, Underwood JC, Karajeh MA, et al Long-term prognostic significance of persisting histological activity despite biochemical remission in autoimmune hepatitis. Am J Gastroenterol 2015, 110, 993–9. 26010310 10.1038/ajg.2015.139

[55] Renand A, Habes S, Mosnier JF, Aublé H, Judor JP, Vince N, et al Immune alterations in patients with type 1 autoimmune hepatitis persist upon standard immunosuppressive treatment. Hepatol Commun 2018, 2, 968–981. 30094407 10.1002/hep4.1202 PMC6078209

[56] Burton OT, Bricard O, Tareen S, Gergelits V, Andrews S, Biggins L, et al The tissue-resident regulatory T cell pool is shaped by transient multi-tissue migration and a conserved residency program. Immunity 2024, 57, 1586–602.e10. 38897202 10.1016/j.immuni.2024.05.023

[57] Shevyrev D, Tereshchenko V. Treg heterogeneity, function, and homeostasis. Front Immunol 2020, 10, 3100. 31993063 10.3389/fimmu.2019.03100 PMC6971100

[58] Dyson JK, Wong LL, Bigirumurame T, Hirschfield GM, Kendrick S, Oo YH, et al Inequity of care provision and outcome disparity in autoimmune hepatitis in the United Kingdom. Aliment Pharmacol Ther 2018, 48, 951–60. 30226274 10.1111/apt.14968 PMC6667893

