# Supplementary material for: An imbalance between CD80 and CD86 levels and CD4 regulatory T cell number and transendocytosis function exists in the liver in autoimmune hepatitis
Source: Clin Exp Immunol. 2026 Mar 11;220(1):uxag013. doi: 10.1093/cei/uxag013 (PMC13034551; doi:10.1093/cei/uxag013)
Supplement: uxag013_Supplementary_Data [file uxag013_supplementary_data.pdf]

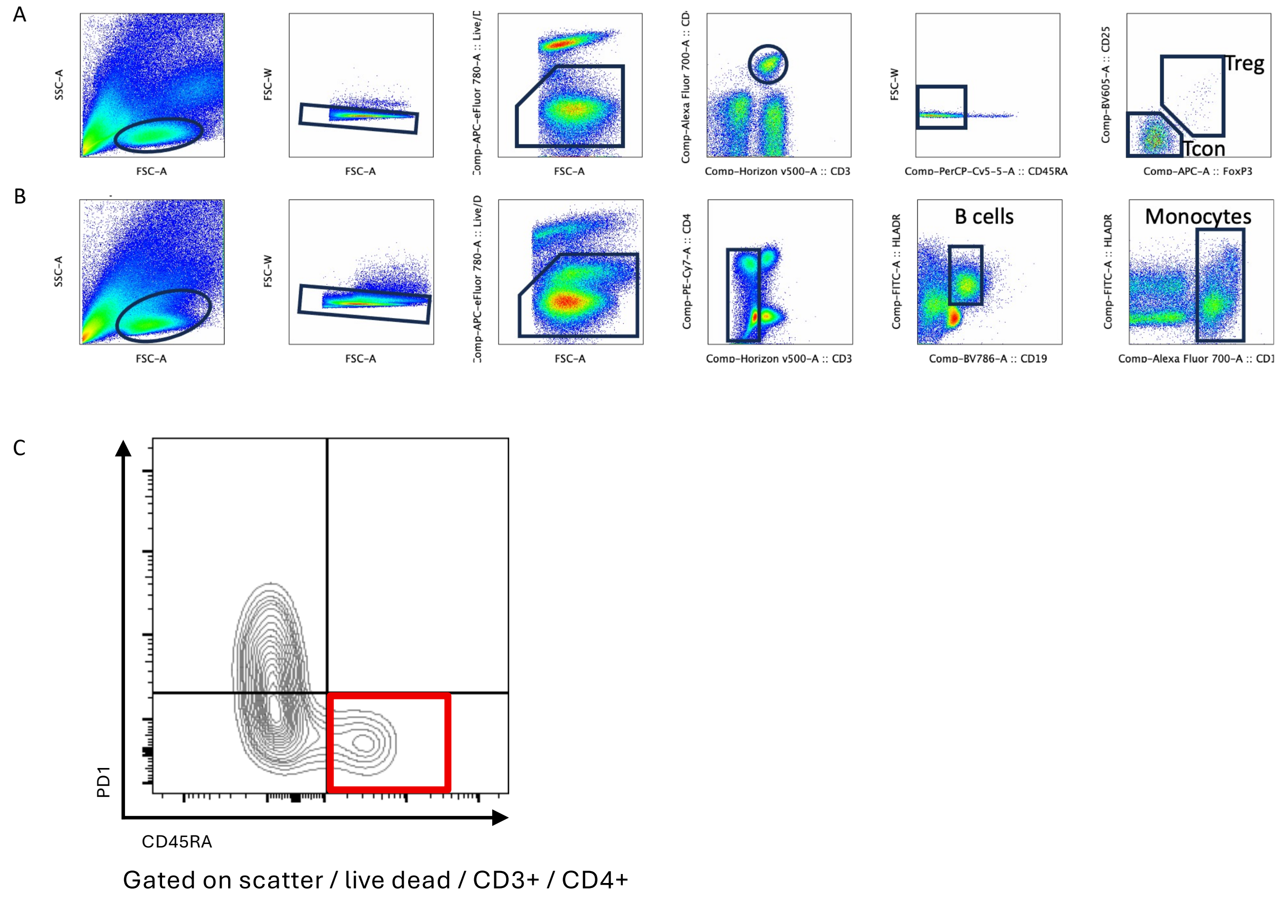

Supplementary Figure 1: gating strategy from representative sample of intrahepatic mononuclear cells for identification of **A** – Tcon and Treg, and **B** – B cells and monocytes. **C** – Representative flow cytometry plot of CD3+CD4+ T lymphocytes isolated from human liver, illustrating how populations of cells which did not stain for markers were identified. In this example, the CD45RA+ population of cells do not express PD1 (gate in lower right quadrant) and therefore the fluorescence intensity cut off between PD1 positive and negative populations can be defined, and a negative population median fluorescence intensity can be derived to allow for internal normalization, if required. Similar approaches were used for all markers assessed where needed.

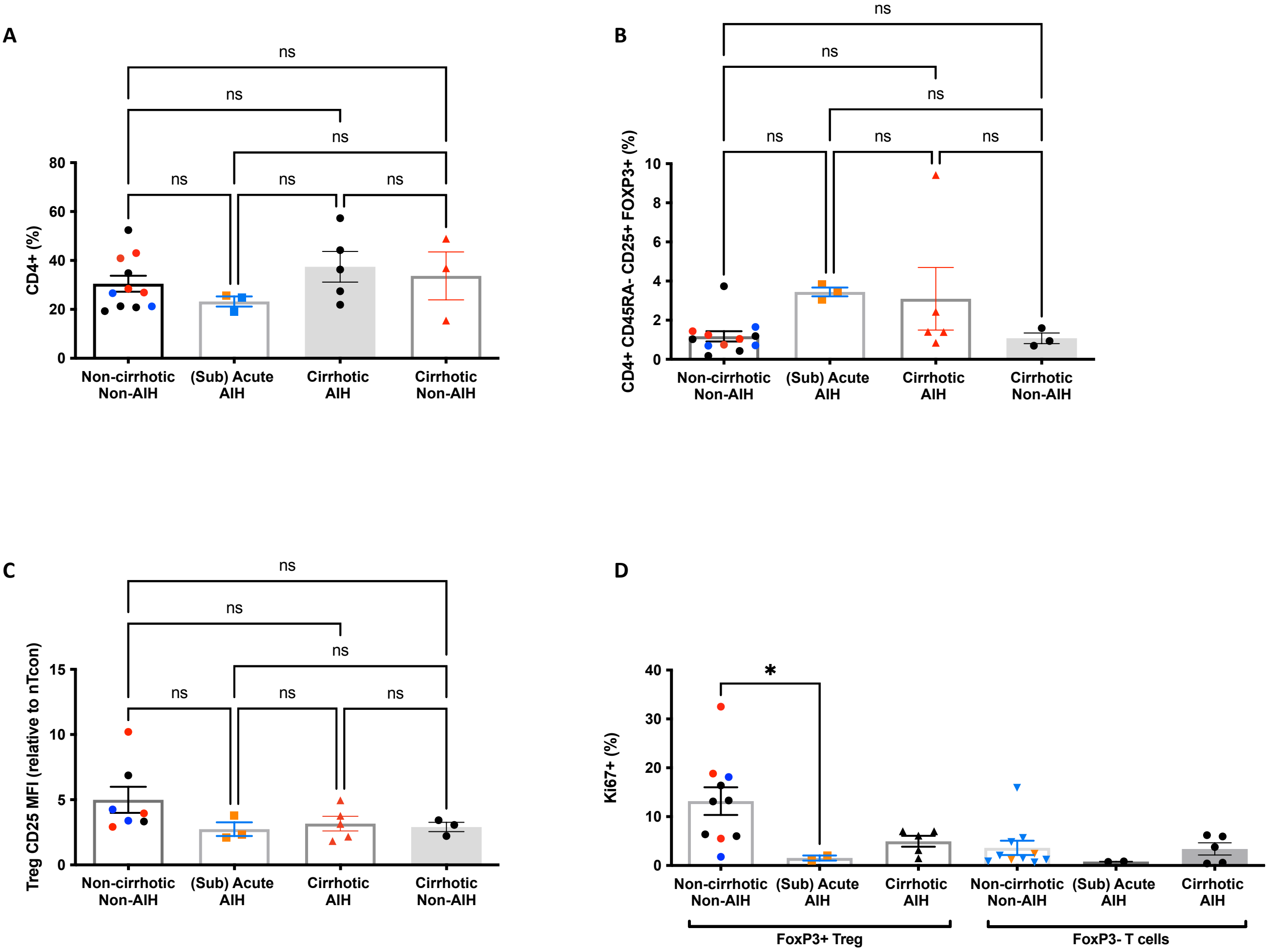

Supplementary Figure 2: Comparison of T cell frequency and proliferation in liver tissue comparing patients with acute AIH, chronic AIH with cirrhosis and tissue from non-cirrhotic non-AIH liver and cirrhotic non-AIH liver. A: A: CD4+ T cell frequency within the CD3+ T lymphocyte population B: Comparison of Treg frequency, demonstrating the percentage of CD4+CD45RA-CD25+FoxP3+ within CD3+ T lymphocyte population. C: CD25 MFI in Treg populations D: Ki67 expression. Graphs show aggregate data for all samples tested. ns = non-significant, * $p < 0.05$ by one way ANOVA with Tukey's multiple comparison test or by unpaired t-test for comparisons of 3 or 2 groups, respectively.

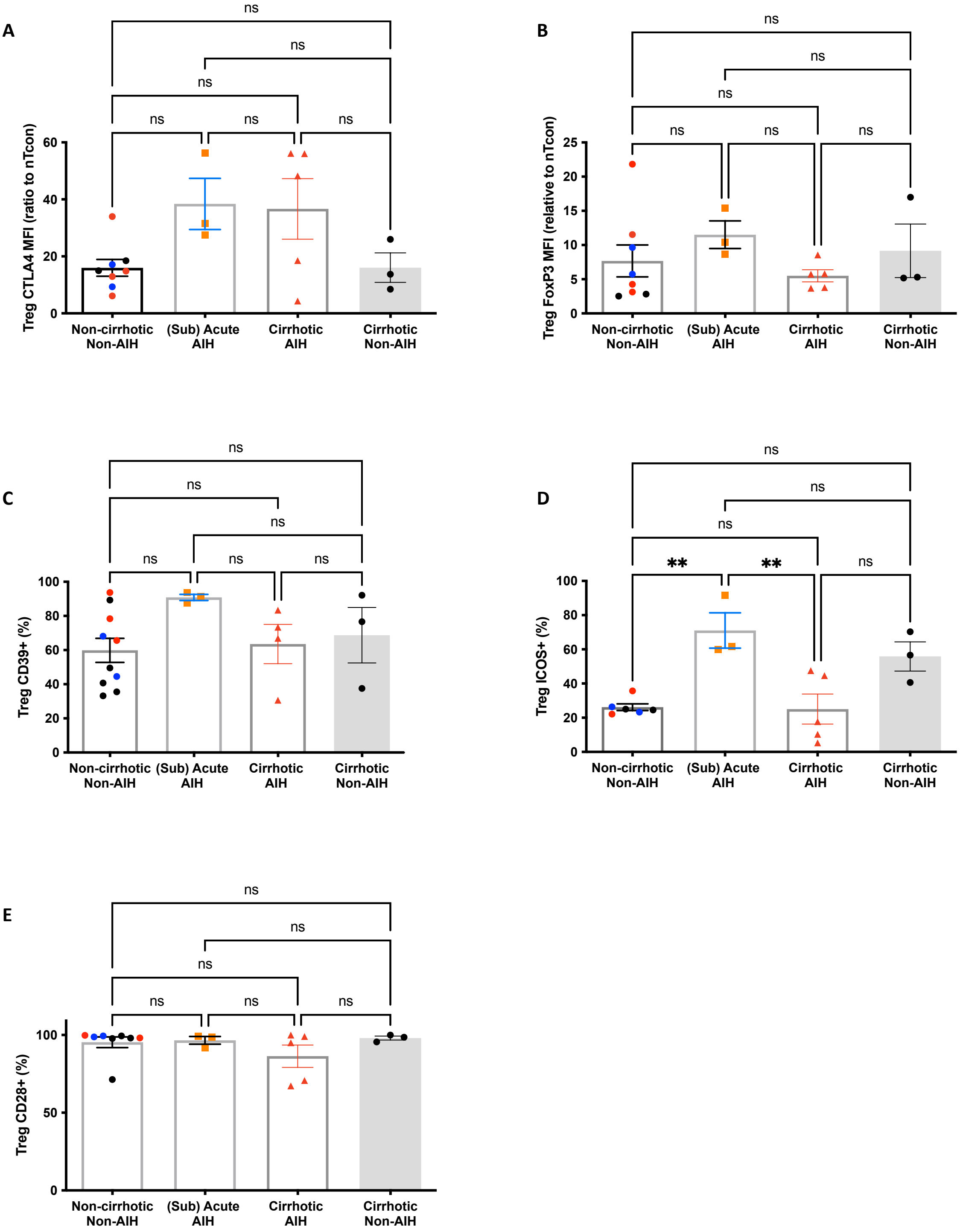

Supplementary Figure 3: Comparison of the phenotype of CD4+CD45RA-CD25+FoxP3+ Treg from liver tissue comparing patients with acute AIH, chronic AIH with cirrhosis and tissue from non-cirrhotic non-AIH liver and cirrhotic non-AIH liver. A: CTLA4 B: FoxP3 C: CD39 D: ICOS E: CD28 expression. Graphs show aggregate data for all samples tested with MFI normalized to an internal negative population (naïve Tcon (CD3+CD4+CD45RA+FoxP3-)) where stated. ns = non-significant, * $p < 0.05$, ** $p < 0.01$ by one way ANOVA with Tukey's multiple comparison test.

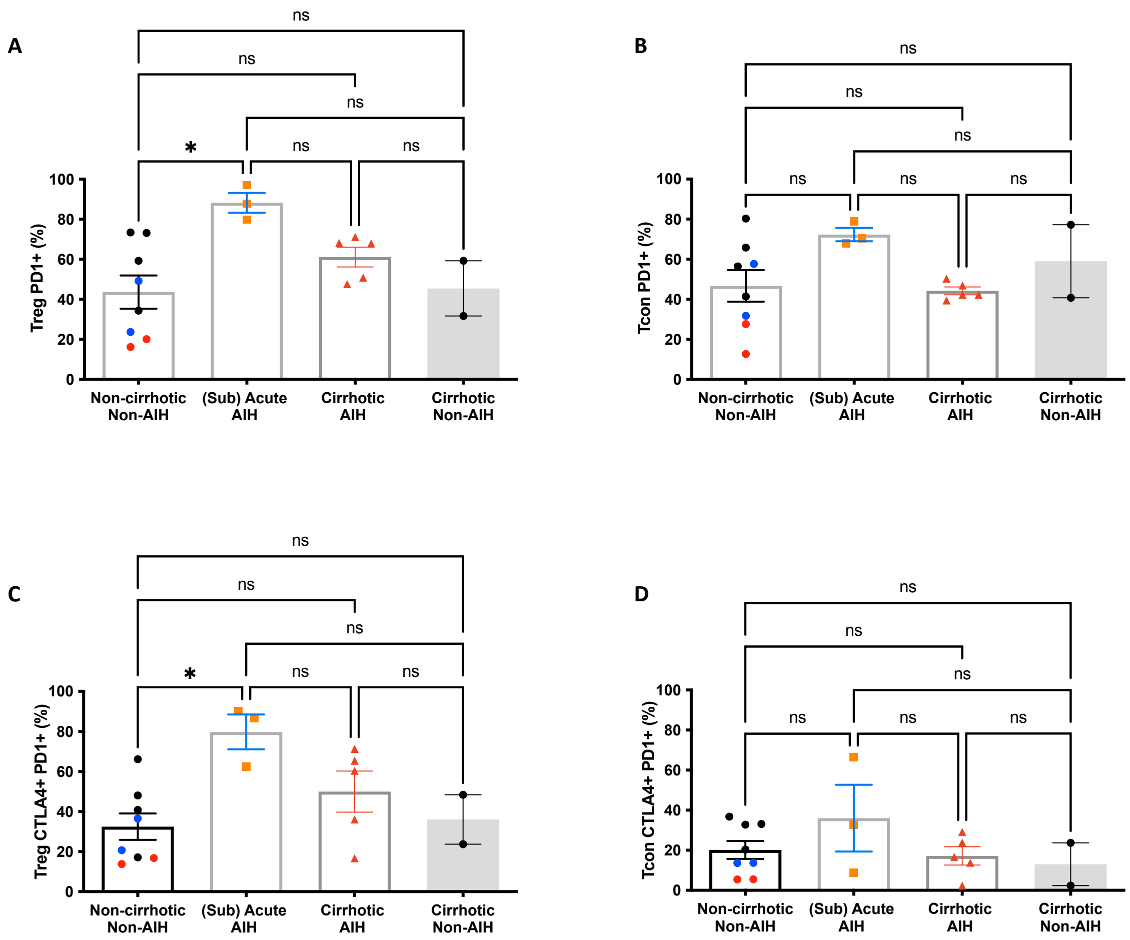

Supplementary Figure 4: Comparison of Treg and Tcon CTLA4 and PD1 expression from liver tissue comparing patients with acute AIH, chronic AIH with cirrhosis and tissue from non-cirrhotic non-AIH liver and cirrhotic non-AIH liver. A: CD4+CD45RA-CD25+FoxP3+ Treg and B: CD4+CD45RA-FoxP3- memory Tcon frequency of PD1+ cells C: CD4+CD45RA-CD25+FoxP3+ Treg and D: CD4+CD45RA-FoxP3- memory Tcon frequency of CTLA4+PD1+ subsets. Graphs show aggregate data for all samples tested. ns = non-significant, * $p < 0.05$, by one way ANOVA with Tukey's multiple comparison test.

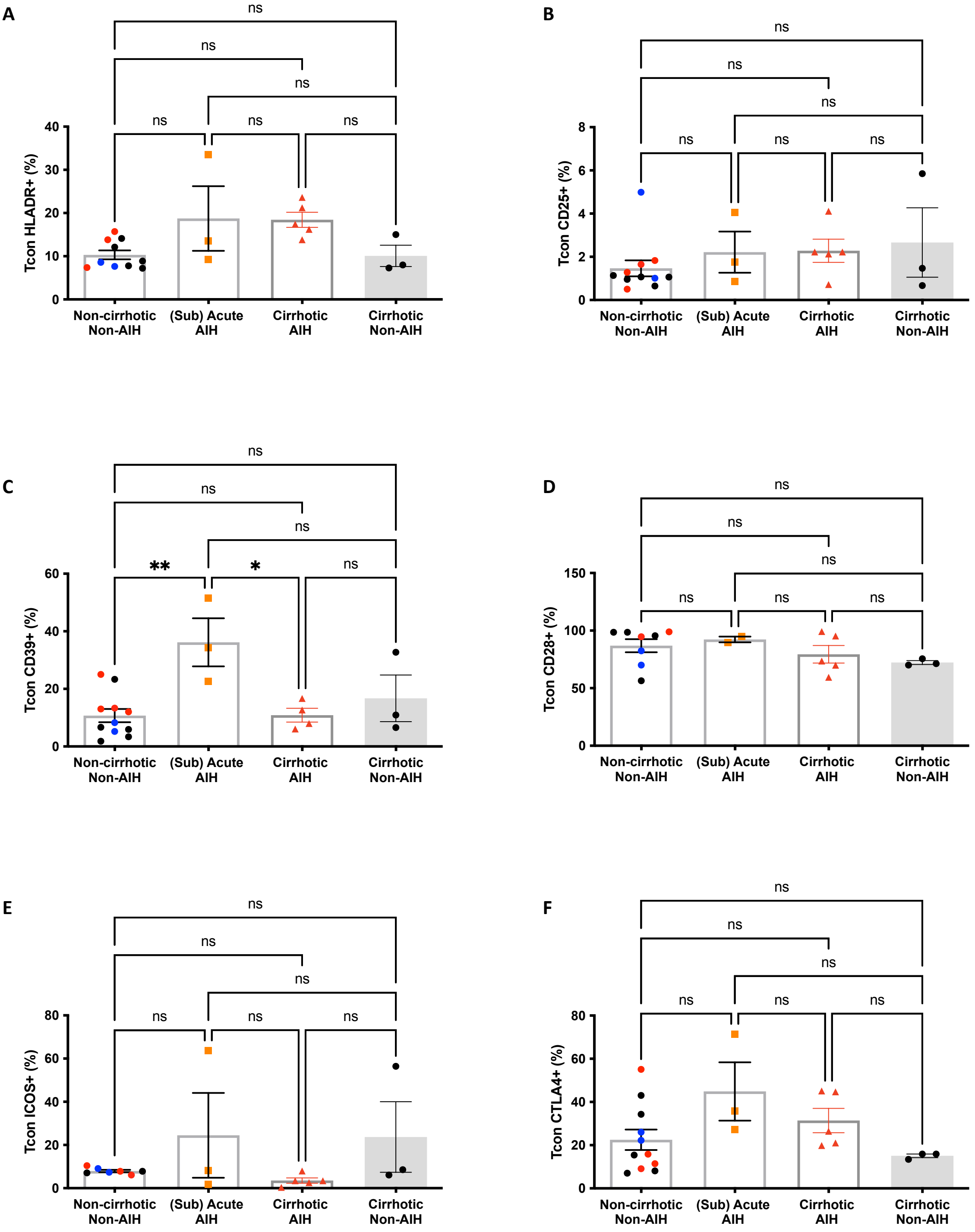

Supplementary Figure 5: CD4+CD45RA-FoxP3- memory Tcon phenotype comparing liver tissue from patients with acute AIH, chronic AIH with cirrhosis and tissue from non-cirrhotic non-AIH liver and cirrhotic non-AIH liver. Frequency of A: HLA-DR expression B: CD25 C: CD39 D: CD28 E: ICOS and F: CTLA4 expression. Graphs show aggregate data for all samples tested. ns = non-significant, * $p < 0.05$, ** $p < 0.01$ by one way ANOVA with Tukey's multiple comparison test.

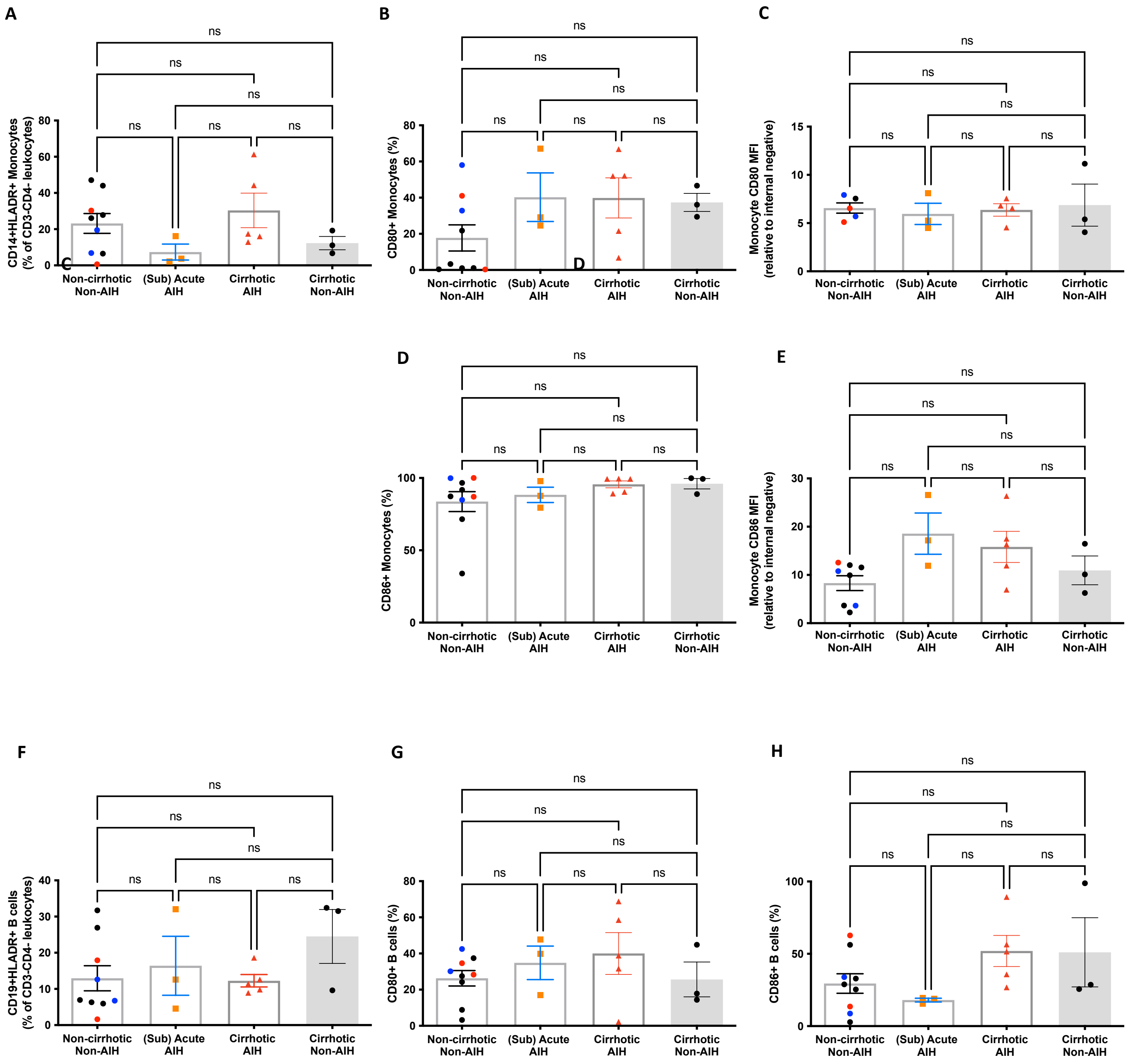

Supplementary Figure 6: Comparison of intrahepatic monocyte and B cell populations between patients with acute AIH, chronic AIH with cirrhosis and tissue from non-cirrhotic non-AIH liver and cirrhotic non-AIH liver. A: frequency CD14+HLA-DR+ monocytes. B: frequency of CD80+ C: CD80 MFI D: frequency of CD86+ and E: CD86 MFI in monocytes. F: frequency CD19+HLA-DR+ B cells G: frequency of CD80+ and H: frequency of CD86+ B cells. Graphs show aggregate data for all samples tested with MFI normalized to an internal negative population (CD80/CD86 negative CD3+ T lymphocytes) where stated. ns = non-significant by one way ANOVA with Tukey's multiple comparison test.
